# Integrated management of fruit trees and *Bletilla striata*: implications for soil nutrient profiles and microbial community structures

**DOI:** 10.3389/fmicb.2024.1307677

**Published:** 2024-03-06

**Authors:** Qiufeng Xie, Huimei Xu, Rouyuan Wen, Le Wang, Yan Yang, Haizhu Zhang, BaoShun Su

**Affiliations:** ^1^College of Pharmaceutical Science, Dali University, Dali, China; ^2^Western Yunnan Traditional Chinese Medicine and Ethnic Drug Engineering Center, College of Pharmacy, Dali University, Dali, China; ^3^Dali Lin Yun Biotechnology Development Co., Ltd., Dali, China

**Keywords:** agroforestry integration, rhizosphere soil, soil physicochemical properties, microbial community, *Bletilla striata*

## Abstract

**Introduction:**

Forest medicinal compound systems in agroforestry ecosystems represent a multi-layered cultivation approach that utilizes forest resources efficiently. However, research on how these systems affect soil nutrients and microbial communities is limited.

**Methods:**

This study compared the soil chemical properties and microbial communities of *Bletilla striata* (C) grown alone versus in agroforestry systems with apple (PB), pear (LB), and peach trees (TB), aiming to understand the impact of these systems on soil health and microbial diversity.

**Results:**

Soil in the GAB systems showed increased levels of essential nutrients but lower pH and ammonium nitrogen levels compared to the control. Significant improvements in organic matter, total phosphorus, and total potassium were observed in TB, PB, and LB systems, respectively. The bacterial diversity increased in GAB systems, with significant changes in microbial phyla indicative of a healthier soil ecosystem. The correlation between soil properties and bacterial communities was stronger than with fungal communities.

**Discussion:**

Integrating *B. striata* with fruit trees enhances soil nutrients and microbial diversity but may lead to soil acidification. Adjustments such as using controlled-release fertilizers and soil amendments like lime could mitigate negative impacts, improving soil health in GAB systems.

## 1 Introduction

*Bletilla striata*, initially recorded in the “Shennong’s Classic of Materia Medica,” is also referred to as the windbell orchid, windbell *Bletilla*, and Wulan (in China). It is generally known as a species under the *Bletilla* genus of the Orchidaceae family. Its tuber is frequently used for medicinal purposes (Sun B. et al., 2022; Zhu et al., 2023). The primary components include polysaccharides, phenanthrenes, phenanthroquinones, bibenzyls, triterpenes, lignans, and organic acids, showcasing a range of biological activities such as anti-inflammatory (Jiang et al., 2019; Zhang C. et al., 2021), antifibrotic (Jiang et al., 2023), anti-tumor (Liu et al., 2022), and immune-regulatory effects (Gong et al., 2023), thereby highlighting its significant medicinal value. Furthermore, due to the vivid and diverse colors of *B. striata* flowers, their prolonged blooming period, and strong adaptability, they have commendable applications in horticulture. With the escalating market demand for *B. striata*, its large-scale production has become prominent (Han et al., 2023). In situations where forest resources are limited, singular cultivation practices often do not maximize the utilization of light, water, and soil resources within the same three-dimensional space. A monotonous plant community structure can lead to population degradation, continuous cropping barriers, allelopathic suppression, and other challenges, resulting in a weakened ecological function of the cultivated area (Zhang H. et al., 2020; Yang et al., 2023). In light of these challenges, it is imperative to adopt effective technical strategies to enhance the economic benefits farmers gain from cultivating *B. striata*, further promoting its sustainable development.

The forestry-medicated intercropping system seamlessly integrates the understorey space, forest canopy shading, and unique climatic and soil characteristics, leading to a notable enhancement in forest land productivity and economic yield. This approach not only bolsters the ecological diversity and soil health of the forested region but also fortifies the stability of the forestry-medicated ecosystem. With the growing emphasis on combined cultivation methods, forestry-medicated intercropping has gradually been recognized as a mainstream cultivation strategy. Such a system not only fosters the efficient utilization of plant resources and space but also plays a pivotal role in the continuous improvement of the soil environment. Its immense potential to enhance ecological diversity, optimize the ecological environment, and offer a myriad of ecosystem services hints at its broad application prospects (Mortimer et al., 2018; Wolz and DeLucia, 2018; Zhang et al., 2018). Several instances of forestry-medicated composite systems have been documented; for example, Verma et al. (2013) discerned that a combined management system of forestry and medicinals facilitated the accumulation of active medicinal components in mint. Research by Sujatha and Bhat (2010) revealed that intercropping vanilla orchids in betel nut gardens augments soil pH, quick-acting soil nutrient content, and economic returns per unit area. In recent years, an increasing number of scholars have opined that studies on crop growth, development, and yield formation should pivot back to the rhizosphere (Philippot et al., 2013). The forestry-medicated intercropping pattern has been proven to safeguard the soil, enrich soil organic matter (OM) and nutrients, amplify biodiversity, bolster ecosystem stability, disrupt pest life cycles, and thereby curtail reliance on chemical pesticides (Wang et al., 2020). While rational forest land combined management can enhance soil fertility (Li B. et al., 2022), not all underforest-combined operations achieve synergistic benefits. For instance, Cardoso et al. (2003) found that the phosphorus content in soil from single-cropped coffee was higher than in its intercropped counterpart. Currently, research on *B. striata* primarily focuses on its intercropping with *Phyllostachys pubescens*. After intercropping *B. striata* with *P. pubescens*, the soil organic carbon (SOC), available nitrogen (AN), and available phosphorus (AP) significantly surged, enhancing microbial abundance. Additionally, there was a significant increase in the total phenol and flavonoid content in *B. striata* (Deng et al., 2023). Intercropping *B. striata* augments soil quality and medicinal material standards.

While some studies have delved into alterations in soil microbial traits induced by forestry-medicinal intercropping (Lin et al., 2012), detailed investigations into shifts in the soil microbial community resulting from the combined management of *B. striata* with fruit trees remain uncharted territory. Therefore, a comprehensive understanding of the soil microbial communities within these *B. striata* agroforestry-based (GAB) systems could be instrumental in the design and management of forestry-medicinal intercropping systems. In this study, we embarked on assessing the changes occurring within bacterial and fungal microbiomes in the context of *B. striata* intercropped with a diversity of fruit trees. We postulate that forestry-medicinal intercropping can significantly modulate the physicochemical properties of the soil, influence the diversity of its microbial communities, and induce shifts in the structure of these communities. The primary objectives of this research are twofold: to analyze variations in the structure of soil microbial communities under different *B. striata*-fruit tree intercropping paradigms using high-throughput sequencing techniques. To elucidate the correlations between these microbial community structures and soil environmental factors under various *B. striata*-fruit tree combined management scenarios, thereby offering theoretical underpinning for the scientific establishment of *B. striata* forestry-medicinal intercropping models.

## 2 Materials and methods

### 2.1 Experimental materials and site description

The research site is located in Dacheng Village, Wase Town, Dali City, Yunnan Province, at the Dali Linyun Tourist Garden in Fengweiqing (100°29′19″E, 25°90′67″N; altitude 2,332 m). The field soil is yellow soil, and the garden has been continuously cropping *B. striata* for 3 years. This region is characterized by a North subtropical highland monsoon climate, with mild weather, no extreme heat or severe cold, minimal wind frost, and spring-like conditions throughout the year. The average annual temperature is 15.4°C, with an average annual rainfall of 510 mm.

**FIGURE 1:**
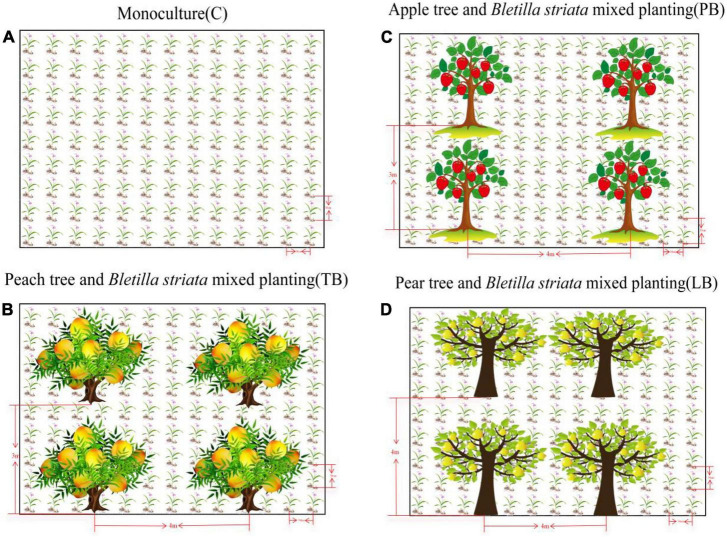
Schematic representation of *Bletilla striata* cultivation patterns. **(A)** Monoculture of *B. striata* (C); **(B)** *B. striata* intercropped with Peach trees (TB); **(C)** *B. striata* intercropped with Apple trees (PB); **(D)** *B. striata* intercropped with Pear trees (LB).

Peach trees (*Prunus persica*), Pear trees (*Pyrus sorotina*), and Apple trees (*Malus pumila* Mill.) are intercropped with *B. striata*. The experimental design includes four treatments: (1) Peach trees – *B. striata* intercropping (TB); (2) Pear trees – *B. striata* intercropping (LB); (3) Apple trees – *B. striata* intercropping (PB); (4) Solo cultivation of *B. striata* (C). The field experiment employs a completely randomized design, with three replicates for each treatment (each plot being 100 m^2^). The agroforestry compound planting garden was established in August 2014, and *B. striata* was planted in April 2019. The planting densities for different treatments are as shown in Figure 1. All crops are irrigated by drip irrigation, and the cultivation process is managed routinely.

In October 2022, soil from the rhizosphere of *B. striata* in each plot was collected. Five soil sub-samples were randomly collected from each plot, mixed to form one root sample, and repeated three times. After removing debris such as stones and residual roots, the soil samples were sealed in pre-prepared sterile bags, quickly transported back to the laboratory in ice boxes, and sieved through a 2 mm soil sieve. The samples were then mixed evenly and divided into two portions. One portion was stored in a −80°C freezer for DNA extraction, and the other portion was air-dried for analysis of soil physicochemical properties.

### 2.2 Determination of soil physicochemical properties

The rhizosphere soil was air-dried at 25°C, subsequently ground, and then sieved through a 0.84 mm mesh. Parameters such as soil pH, OM, total nitrogen (TN), total phosphorus (TP), total potassium (TK), AN, readily available phosphorus (AP), available potassium (AK), NH_4_^+^-N, and nitrate nitrogen (NO_3_^–^-N) were measured. The soil pH was determined using a PHS-3C pH meter (Shanghai Scientific Instrument Co. Ltd., Shanghai, China), Precisely weigh 10.00 g of soil sample and place it in a 50 ml centrifuge tube. Add 25 ml of distilled water, shake vigorously for 2 min, and let it stand for 30 min to measure the pH. The determination of OM, TN, TP, TK, AN, AP, AK, NH_4_^+^-N, and NO_3_^–^-N was carried out following the methods described in the third edition of “Soil Agrochemical Analysis,” edited by Bao (2000). The book, published by the China Agricultural Press in 2000, is widely regarded as an authoritative source in the fields of soil science and agriculture. We chose to follow its methods because they are not only widely applied in Chinese soil analysis practices but also align with internationally accepted standards in their fundamental principles and procedural steps. Despite differences in specific chemicals and equipment used, by incorporating modern measurement technology from the LD-GT1 Intelligent Soil Nutrient Analyzer (Shandong Lainde Intelligent Technology Co., Ltd., Shandong, China), we were able to execute these methods with greater efficiency and accuracy. Additionally, the LD-GT1 employs advanced sensing technology and automated processes for the effective quantification of OM content in soil, a methodology whose accuracy has been corroborated through scholarly research and practical application (Pu et al., 2022). The determination of OM was carried out using the potassium dichromate dilution hot colorimetric method. The procedure is as follows: initially, a bag of soil OM extraction agent powder was placed in a 500 ml plastic bottle, and distilled water was added up to the marked line to prepare the soil OM extraction solution. Subsequently, 4.0 g of air-dried soil sample was weighed and added to the extraction bottle, followed by the addition of 20 ml of the aforementioned extraction agent. The mixture was thoroughly shaken and agitated for 5 min before filtration. The resultant filtrate served as the test solution for OM. The determination of AN employed the alkali digestion distillation method. Precisely, 1.0 g of air-dried soil sample was placed in a 100 ml conical flask, followed by the sequential addition of 25 ml distilled water, 12 drops of hydrolysis agent, 2 drops of stabilizer, and 1.0 g of reductant (a mixture of ferrous sulfate and zinc powder in a 5:1 ratio). The conical flask was immediately sealed and connected to an absorption bottle containing distilled water (with 3 drops of absorbent) via a distillation tube. The flask was then placed on an asbestos net and heated with an alcohol lamp, causing the mixture to boil and sustain distillation for 7 min. After cooling, the liquid in the absorption bottle was transferred to a 100 ml volumetric flask, and the apparatus was rinsed with distilled water before being brought up to volume. The resulting solution was the test solution for AN. The determinations of NH_4_^+^-N, NO_3_^–^-N, AP, and AK in the soil were conducted respectively using the Nessler’s reagent colorimetric method, nitrate test powder method, molybdenum blue colorimetric method, and tetraphenylborate sodium turbidimetric method. A bag of combined soil extraction agent powder was added to a 500 ml volumetric flask and brought up to volume with distilled water to prepare the soil extraction agent. Then, 1.0 g of air-dried soil sample was weighed and added to an extraction bottle along with 20 ml of soil extraction agent and an appropriate amount of soil decolorizing agent. After vigorous shaking for 3 min and subsequent filtration, the filtrate obtained served as the test solution for NH_4_^+^-N, NO_3_^–^-N, AP, and AK. Furthermore, the contents of TN, TP, and TK in the soil were determined using the Kjeldahl distillation method, acid dissolution-antimony molybdenum colorimetric method, and perchloric acid-nitric acid digestion method, respectively. Similarly, a bag of combined soil extraction agent powder was added to a 500 ml volumetric flask and made up to volume with distilled water. Subsequently, 1.0 g of air-dried soil sample was placed in a Kjeldahl flask, moistened with 10 drops of water, followed by the sequential addition of 0.2 g of TN reductant, 4 ml of concentrated sulfuric acid, and 10 drops of soil TN oxidant. After heating, the contents of the Kjeldahl flask were transferred to a 100 ml volumetric flask and made up to volume with distilled water. Finally, 20 ml of the clear supernatant was transferred to another 100 ml volumetric flask, to which 1.6 ml of TN regulator (1:1 NaOH solution) was added, and then brought up to volume with distilled water and filtered. The resultant filtrate served as the test solution for TN, TP, and TK. During all determinations, 2 ml of distilled water, 2 ml of soil extraction solution (containing 1 drop of soil standard reserve solution), and 2 ml of soil test solution were used as the blank, standard, and test solutions, respectively. These samples were placed in three separate test tubes, followed by the addition of respective soil testing reagents. After thorough mixing and standing, the solutions were transferred to cuvettes for the determination of nutrient contents.

### 2.3 Soil DNA extraction, PCR amplification, and sequencing

Weigh 0.5 g of fresh soil and extract DNA from the samples using the CTAB method, repeating the process three times. The DNA amplification products are then detected using the Agilent 5400 automatic capillary electrophoresis system to assess the quality and concentration of DNA. Subsequently, an appropriate amount of sample DNA is taken in a centrifuge tube and diluted with sterile water to 1 ng/μl. PCR amplification of the bacterial V3–V4 region is performed using primers 341F (5′-CCTAYGGGRBGCASCAG-3′) and 806R (5′-GGACTACNNGGGTATCTAAT-3′); and for fungal ITS fragments, primers ITS1-1F-F (5′-CTTGGTCATTTAGAGGAAGTAA-3′) and ITS1-1F-R (5′-GCTGCGTTCTTCATCGATGC-3′) are used. The reaction system includes 10 ng of gDNA template, 15 μl of Phusion Master Mix (2×), 0.2 μl of each upstream and downstream primer (1 μM), and ddH_2_O added up to 30 μl. The amplification program is as follows: initial denaturation at 98°C for 1 min; followed by 30 cycles of 98°C denaturation for 10 s, 50°C annealing for 30 s, 72°C extension for 30 s; and a final extension at 72°C for 5 min. The PCR products are extracted from a 2% agarose gel and purified and quantified using the Qiagen gel recovery kit (Qiagen, Germantown, MD, USA) along with Qubit fluorometric quantitation and Q-PCR methods. High-throughput sequencing is conducted using the Illumina NovaSeq6000 platform.

### 2.4 Sequencing data processing

Based on Barcode and PCR amplification primer sequences, individual sample data were extracted from the initial sequencing output. Following the removal of Barcode and primer sequences, FLASH (v1.2.11^1^) was employed to assemble reads for each sample, yielding the primary Raw Tags data. These assembled Raw Tags then underwent rigorous filtration to derive high-quality Clean Tags data. Adhering to the Tags quality control process outlined by Qiime (V1.9.1^2^), the following steps were executed: (a) Tags Truncation: Raw Tags were truncated at the first low-quality base position when a predefined length (default value set to 3) of consecutive bases exhibited a quality threshold of ≤ 19; (b) Tags Length Filtering: following truncation, the resulting Tags dataset was further refined to exclude Tags with high-quality base lengths less than 75% of the total Tags length. After these procedures, the resultant Tags underwent chimera sequence removal. The Tags sequences were aligned with species annotation databases via VSEARCH^3^ to detect chimeric sequences, which were subsequently removed to produce the final Effective Tags.

### 2.5 Statistical data analysis

Unless otherwise specified, data statistics and visualization were conducted using the R programming language and its associated packages. Based on the experimental design, the acquired OTU data was filtered, removing OTUs with a prevalence of less than 30% in soil samples. After standardizing the OTU table using the TMM (Trimmed Means of M) algorithm, the relative expression abundance of OTUs was calculated. The vegan package was employed to determine microbial α-diversity and β-diversity (utilizing the Shannon and Chao1 indices). One-way analysis of variance (ANOVA) and the least significant difference (LSD) method (*P* < 0.05) were performed using SPSS 21.0 (IBM, USA) to ascertain if the differences in diversity between samples based on α and β indices were statistically significant. The “ggplot2” package facilitated the principal coordinate analysis (PCoA) to assess the distribution trends of soil microbial communities under different treatments. Similarity analysis (ANOSIM) and permutationl multivariate analysis of variance (ADONIS) with 999 permutations were used to identify significant inter-sample group differences in β-diversity based on the Bray–Curtis distance matrix. Linear discriminant analysis Effect Size (LEfSe) was utilized to elucidate taxonomic features characterizing inter-treatment microbial species differences.^4^ To simplify the LEfSe workflow, OTU table filtering was conducted, selecting only OTUs with a relative abundance of *P* > 0.01%. The factorial Kruskal–Wallis sum-rank test (α = 0.05) was employed to identify taxa with significant group inter-abundance differences. Subsequently, the effect size of each discriminative trait was calculated based on log LDA scores (threshold = 4.0). The Spearman rank correlation test was applied to determine correlations between module feature genes and soil properties. The “vegan” package was utilized for redundancy analysis (RDA) to explore the relationships between environmental factors, dominant microbes, and microbial functions. The Spearman rank correlation test further examined the explanatory power of environmental factors on microbial species and functionalities.^5^

## 3 Results

### 3.1 Effects of intercropping on soil physicochemical properties

In all orchard-*B. striata* integrated planting soil systems (LB, TB, and PB), levels of TN, AN, AP, and AK were significantly higher than in monoculture systems (C) (LSD, *P* < 0.05, Table 1). Compared to C, the content of TN, AN, AP, AK, and NO_3_^–^-N in LB increased by 22.37%, 34.31%, 163.49%, 146.22%, and 63.90%, respectively. In PB, the levels of TN, AN, AP, and AK were elevated by 13.70%, 49.44%, 120.86%, and 147.12% in comparison to C. For TB, TN, AN, AP, AK, and NO_3_^–^-N content increased by 6.85%, 64.91%, 161.71%, 106.53%, and 16.82% relative to C (Table 1). Among the different intercropping soil systems, the soil in LB showed the highest concentrations of TN, TK, AP, and AK. The soil in PB had the highest levels of TP and AK, while the soil in TB presented the maximum concentrations of OM, AN, and AP (Table 1). The soil pH values in the LB, PB, and TB systems were significantly different from C, all showing a marked reduction (Table 1).

**TABLE 1:** Chemical properties of the soil in the *Bletilla striata* rhizosphere.

	C	LB	PB	TB	*P*-values	Significant
OM	167.7 ± 0.49b	167.07 ± 0.60b	164.49 ± 1.15c	185.04 ± 0.35a	0	***
TN	2.19 ± 0.02d	2.68 ± 0.04a	2.49 ± 0.02b	2.34 ± 0.03c	0	***
TP	0.84 ± 0.01b	0.76 ± 0.05b	1.09 ± 0.07a	0.66 ± 0.03c	0	***
TK	146.54 ± 4.23b	177.17 ± 7.96a	117.19 ± 5.28d	128.91 ± 2.54c	0	***
AN	143.7 ± 8.83d	193.01 ± 5.41c	214.74 ± 9.95b	236.97 ± 6.05a	0	***
AP	59 ± 1.69c	155.46 ± 1.19a	130.31 ± 1.99b	154.41 ± 1.18a	0	***
AK	196.93 ± 2.56c	484.89 ± 2.71a	486.65 ± 5.61a	406.72 ± 5.77b	0	***
NH_4_^+^-N	96.42 ± 2.31a	5.23 ± 0.98d	34.29 ± 2.32b	19.11 ± 1.15c	0	***
NO_3_^–^-N	72.14 ± 1.25c	118.24 ± 1.50a	66.78 ± 1.33d	86.73 ± 2.48b	0	***
pH	6.8 ± 0.04a	5.69 ± 0.08b	5.44 ± 0.24b	5.69 ± 0.30b	0	***

Values are presented as mean ± SE (*n* = 3). Different lowercase letters indicate statistically significant differences among treatments (*P* < 0.05). The symbol “***” denotes significant differences at the ≤0.001 level. OM, organic matter; TN, total nitrogen; TP, total phosphorus; TK, total potassium; AN, available nitrogen; AP, rapidly available phosphorus; AK, readily available potassium; NH_4_^+^-N, ammonium nitrogen; NO_3_^–^-N, nitrate nitrogen; C, monoculture of *B. striata*; LB, *B. striata* intercropped with Pear trees; PB, *B. striata* intercropped with Apple trees; TB, *B. striata* intercropped with Peach trees.

### 3.2 Impact on *Bletilla striata* root microbial community diversity and structure under different compound modes

**FIGURE 2:**
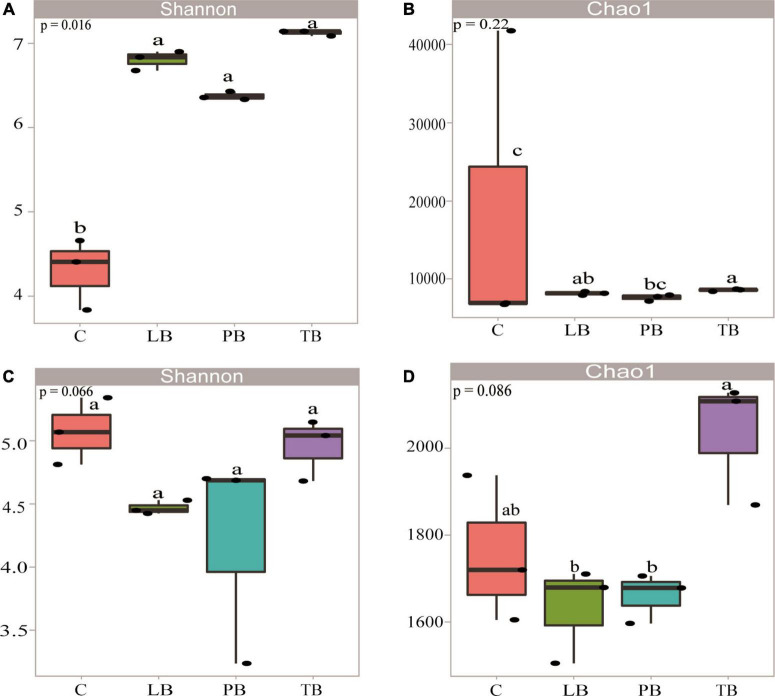
Diversity indices of *Bletilla striata*’s bacterial **(A,B)** and fungal **(C,D)** communities in different intercropping systems. **(A)** Bacterial Shannon diversity index; **(B)** bacterial Chao1 richness index; **(C)** fungal Shannon diversity index; **(D)** fungal Chao1 richness index. Lowercase letters on each box denote the least significant differences (LSD; *P* < 0.05) among treatments. C, monoculture of *B. striata*; LB, *B. striata* intercropped with Pear trees; PB, *B. striata* intercropped with Apple trees; TB, *B. striata* intercropped with Peach trees.

After high-throughput sequencing, approximately 832,533 quality 16s sequences and 818,986 quality ITS2 sequences were produced during the raw-read sequencing process. The average read lengths for bacteria and fungi were 415 and 246 bp, respectively. Using a 97% identity threshold, sequences from all samples were clustered into 9,150 bacterial OTUs and 3,499 fungal OTUs. Figure 2 illustrates the α-diversity indices of bacterial and fungal communities under different intercropping modes, measured based on the Shannon index, encompassing both community richness and evenness. For bacterial microbes, the order of Shannon diversity values was: TB > LB > PB > C. Compared to C, the Shannon diversity values of TB, LB, and PB were significantly elevated (LSD, *P* < 0.05, Figure 2A). The order for Chao1 diversity values was TB > LB > PB > C. In comparison to C, TB and LB exhibited significantly higher Chao1 diversity values (LSD, *P* < 0.05, Figure 2B), while no significant difference was observed between PB and C (*P* > 0.05, Figure 2B). For fungal microbes, the sequence of Shannon diversity values was: C > TB > LB > PB. When contrasted with C, no discernible differences in Shannon diversity indices for TB, LB, and PB were detected (*P* > 0.05, Figure 2C). The order for Chao1 diversity values was TB > C > LB > PB. Relative to C, no significant differences in Chao1 diversity indices were observed for TB, LB, and PB (*P* > 0.05, Figure 2D). However, the Chao1 diversity index for TB surpassed those of PB and LB (*P* < 0.05, Figure 2D).

**FIGURE 3:**
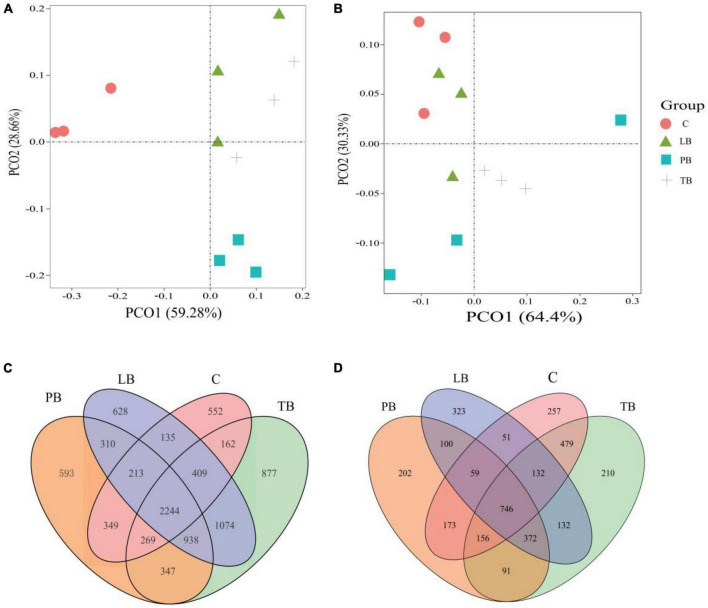
Microbial community diversity under different *Bletilla striata* integrated planting regimes. The principal coordinate analysis (PCoA) plot, based on the Bray–Curtis dissimilarity, illustrates the separation between soil bacterial **(A)** and fungal **(B)** communities across treatments. Venn diagrams display the number of operational taxonomic units (OTUs) for bacteria **(C)** and fungi **(D)** under each intercropping scheme. C, monoculture of *B. striata*; LB, *B. striata* intercropped with Pear trees; PB, *B. striata* intercropped with Apple trees; TB, *B. striata* intercropped with Peach trees.

Assessing microbial community differences among treatments using PCoA based on Bray–Curtis distances. Utilizing the PCoA based on Bray–Curtis distances, we evaluated microbial community distinctions across different treatments. In the context of bacterial microbial communities, clear separations were observed between TB, PB, LB, and C treatments. Notably, LB and PB showcased partial overlaps (Figure 3A). The PCoA results indicated that the first two axes account for 59.28% and 28.66% of the overall variance in bacterial microbial compositions, respectively. Regarding the fungal microbial communities, pronounced separations were discernible between TB, PB, and C, with TB partially overlapping with both C and LB (Figure 3B). The PCoA demonstrated that the first two axes elucidated 64.4% and 30.33% of the total variability in fungal microbial compositions, respectively. Results from ANOSIM revealed significant distinctions in both bacterial and fungal microbial communities between at least two treatments. Additionally, ADONIS results indicated a significant difference in bacterial microbial communities across a minimum of two treatments (Supplementary Table 1). Venn diagrams affirmed that variations in microbial compositions between different soil intercropping systems stemmed from changes in both unique and shared OTU compositions. Out of all identified OTUs in this research, 2,224 bacterial OTUs were common across all intercropping soil systems. Specifically, PB, LB, TB, and C had 593, 628, 877, and 552 unique OTUs, respectively (Figure 3C). For fungal communities, 746 OTUs were shared across all systems, while PB, LB, TB, and C exhibited 202, 323, 210, and 257 unique OTUs, respectively (Figure 3D).

### 3.3 Influence of combined management on soil microbial composition

**FIGURE 4:**
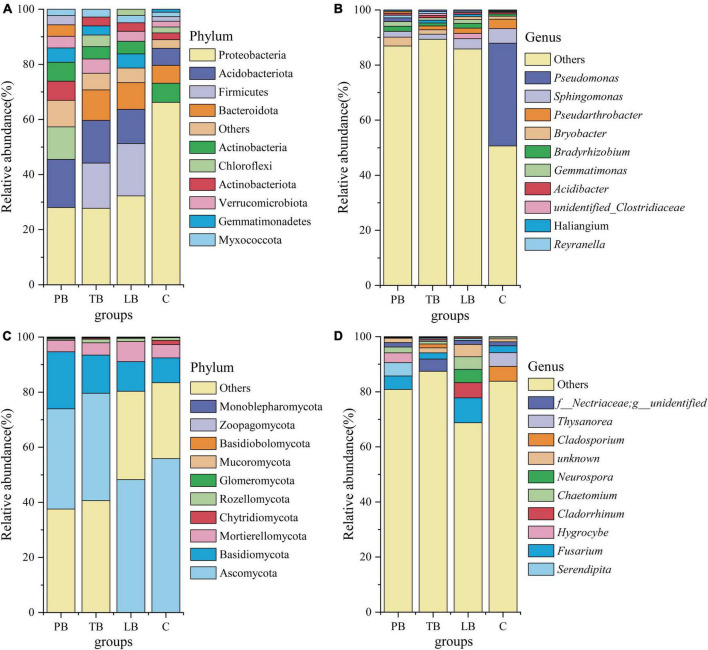
Relative abundance of soil microbial communities at the phylum and genus levels under different intercropping systems. **(A)** Bacterial phylum level; **(B)** bacterial genus level; **(C)** fungal phylum level; **(D)** fungal genus level. C, monoculture of *Bletilla striata*; LB, *B. striata* intercropped with Pear trees; PB, *B. striata* intercropped with Apple trees; TB, *B. striata* intercropped with Peach trees.

Figure 4 presents the results of sequence analyses conducted at both the genus and phylum levels. For bacteria (Figures 4A, B and Supplementary Table 2), the most abundant phylum was Proteobacteria, constituting 38.56%, followed by Acidobacteriota (12.91%) and Firmicutes (12.99%). Among the dominant taxa, no significant variation in abundance was observed in PB’s Myxococcota, Bacteroidota, Actinobacteria, Verrucomicrobiota, and Firmicutes; in TB’s Gemmatimonadetes, Actinobacteria, Verrucomicrobiota, Actinobacteriota, Chloroflexi, and Firmicutes; and LB’s Actinobacteriota, Chloroflexi, Actinobacteria, Verrucomicrobiota, and Acidobacteriota when compared to C (Figure 4A). A notable decline in the abundance of Proteobacteria was evident in PB, TB, and LB compared to C (Figure 4A). Contrarily, an elevated richness was discerned in TB’s Myxococcota, Bacteroidota, and Acidobacteriota; in PB’s Gemmatimonadetes, Actinobacteriota, Chloroflexi, and Acidobacteriota; and LB’s Myxococcota, Bacteroidota, Gemmatimonadetes, and Firmicutes in contrast to C (Figure 4A). The most prevalent genera across different intercropping soils included *Pseudomonas* (9.92%), *Sphingomonas* (3.24%), *Pseudarthrobacter* (1.84%), *Bryobacter* (1.75%), *Bradyrhizobium* (1.36%), and *Gemmatimonas* (1.07%) (Figure 4B). In comparison to the monoculture system (C), the intercropping systems (PB, LB, TB) did not manifest significant shifts in the abundance of *Sphingomonas*, *Pseudarthrobacter*, *Bryobacter*, *Bradyrhizobium*, *Gemmatimonas*, or *Acidibacter*. In juxtaposition with C, TB, and LB showcased a higher abundance of *Haliangium*, whereas LB exhibited a heightened abundance of unidentified *Clostridiaceae*. Furthermore, when contrasted with C, an increased significance was noted in the “others” category across LB, TB, and PB. Conversely, a lower abundance of *Pseudomonas* was evident in PB, TB, and LB when juxtaposed with C.

Referring to fungi, Figures 4C, D and Supplementary Table 3 depict that Ascomycota was the most abundant phylum, making up 44.90% of the samples. It was closely followed by Basidiomycota (13.60%) and Mortierellomycota (5.18%). Among the prevalent taxa, the abundance of Basidiomycota, Mortierellomycota, Zoopagomycota, and Monoblepharomycota remained consistent across the three examined intercropping systems, showing no significant deviation when compared to the monoculture soil system (C) (Figure 4C). In contrast to C, the abundance of Ascomycota and Chytridiomycota in PB, Ascomycota in TB, and Chytridiomycota and Mucoromycota in LB was markedly reduced (Figure 4C). When compared to C, a significantly enhanced abundance of Basidiobolomycota in LB and Glomeromycota in PB was evident (Figure 4C). The most prevalent genera across different intercropping soil systems included *Fusarium* (4.67%), *Nectriaceae* (2.27%), *Chaetomium* (2.03%), *Cladosporium* (1.88%), *Cladorrhinum* (1.57%), *Serendipity* (1.49%), *Thysanorea* (1.37%), *Neurospora* (1.30%), and *Hygrocybe* (1.00%) (Figure 4D). In juxtaposition with the monoculture soil system (C), there was no notable variance in the abundance of *Serendipita*, *Hygrocybe*, and *Chaetomium* across PB, TB, and LB. When contrasted with C, a significant surge in abundance was observed for *Nectriaceae* in TB and *Cladorrhinum* and *Fusarium* in LB. Conversely, a notable decrease in the abundance of *Thysanorea* and *Cladosporium* was evident across PB, TB, and LB compared to C (Figure 4D).

**FIGURE 5:**
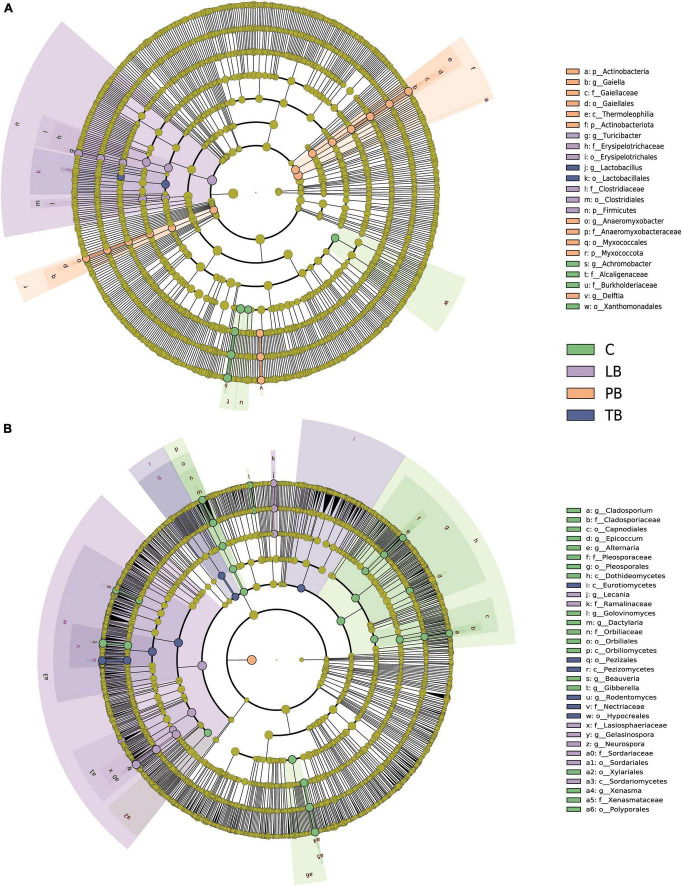
Cladogram from Linear discriminant analysis Effect Size (LEfSe) highlighting the significant discriminative bacterial **(A)** and fungal **(B)** taxa associated with different co-planting schemes (LDA >4). C, monoculture of *Bletilla striata*; LB, *B. striata* intercropped with Pear trees; PB, *B. striata* intercropped with Apple trees; TB, *B. striata* intercropped with Peach trees.

The LEfSe was employed to discern the effects of various composite patterns on the composition of soil microbial communities. The analysis identified significant differences in the relative abundance of 23 bacterial taxa among the different composite soil systems. As shown in Figure 5A, 4 bacterial taxa in C, 6 in LB, 11 in PB, and 2 in TB were pinpointed as biomarkers. Furthermore, the LEfSe delineated that 33 fungal taxa have significant variations in their relative abundance across the different composite soil systems. Notably, the identified biomarkers included 19 fungal taxa in C, 8 in LB, and 6 in TB (Figure 5B).

### 3.4 Relationship between soil properties and soil microbial community

**FIGURE 6:**
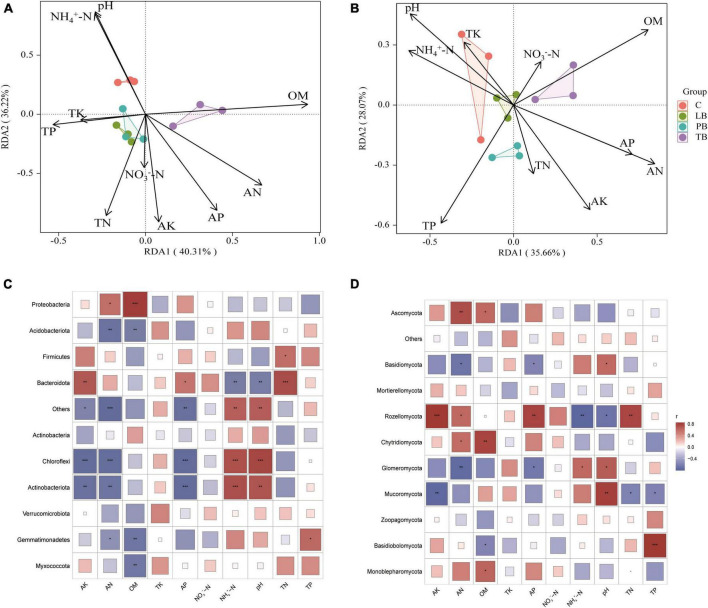
Redundancy analysis (RDA) of bacterial **(A)** and fungal **(B)** community compositions under orchard-*Bletilla strata* combined planting regimes. Statistical correlations between soil physicochemical properties and bacterial **(C)** and fungal **(D)** microbiota. OM, organic matter; TN, total nitrogen; TP, total phosphorus; TK, total potassium; AN, available nitrogen; AP, available phosphorus; AK, readily available potassium; NH_4_^+^-N, ammonium nitrogen; NO_3_^–^-N, nitrate nitrogen; C, monoculture of *Bletilla striata*; LB, *B. striata* intercropped with Pear trees; PB, *B. striata* intercropped with Apple trees; TB, *B. striata* intercropped with Peach trees. *P < 0.05; **P < 0.01; and ***P < 0.001.

To elucidate the effects of climatic factors of different composite modes on fungal and bacterial communities, RDA was employed to correlate dominant species of fungi and bacteria with climatic factors of the composite modes. The results are presented in Figure 6. For bacteria, the RDA results revealed that the RDA1 and RDA2 axes accounted for 40.31% and 36.22% of the variation in the soil bacterial community, respectively, cumulating an overall 76.53% explanation rate (Figure 6A). pH and NH_4_^+^-N were closely associated with C; TP, TK, TN, and AK showed a close relation with LB and PB; while OM, AN, and AP were intimately correlated with TB. Projections of environmental factors onto the first and second ordination axes indicated that AN exhibited the most substantial impact on the bacterial community, followed by pH, OM, TN, AK, AP, and NH_4_^+^-N (*P* < 0.05). TP, NO_3_^–^-N, and TK showed no discernible impact on the bacterial community (Figure 6A and Supplementary Table 4). Concerning fungi, the RDA1 and RDA2 axes represented 35.66% and 28.07% of the variation in the soil fungal community, respectively, amounting to a total explanation rate of 63.73% (Figure 6B). TK, pH, NH_4_^+^-N, and TP were closely tied to both C and LB; TP, TN, and AK correlated strongly with PB; while AP, AN, and OM were linked with TB. Evaluations of environmental factors on the first and second ordination axes identified AN as having the most pronounced influence on the fungal community, trailed by OM, TP, pH, AK, AP, and NH_4_^+^-N (*P* < 0.05). Meanwhile, TN, NO_3_^–^-N, and TK bore no significant effect on the fungal community (Figure 6B and Supplementary Table 4).

To delve deeper into the correlation between dominant microbial species in *B. striata* soil and environmental factors under various composite modes, a Spearman correlation test was conducted between dominant microbial species and environmental factors. For bacteria, as depicted in Figure 6C: Myxococcota was significantly negatively correlated with OM (*P* < 0.01). Gemmatimonadetes showed a positive correlation with TP (*P* < 0.05), a significant negative correlation with OM (*P* < 0.01), and a negative correlation with AN (*P* < 0.05). Actinobacteriota was strongly positively correlated with NH_4_^+^-N (*P* ≤ 0.001), significantly positively correlated with pH (*P* < 0.01), and strongly negatively correlated with AP (*P* ≤ 0.001). It also showed a significant negative correlation with AK and AN (*P* < 0.01). Chloroflexi exhibited strong positive correlations with NH_4_^+^-N and pH (*P* ≤ 0.001), and strong negative correlations with AK, AN, and AP (*P* ≤ 0.001). Bacteroidota was strongly positively correlated with TN (*P* ≤ 0.001), positively correlated with AK (*P* < 0.01), and AP (*P* < 0.05), and significantly negatively correlated with NH_4_^+^-N and pH (*P* < 0.01). Firmicutes showed a positive correlation with TN (*P* < 0.05). Acidobacteriota exhibited a significant negative correlation with AN and OM (*P* < 0.01). Proteobacteria was significantly positively correlated with OM (*P* < 0.01) and positively correlated with AN (*P* < 0.05).

For fungi, as shown in Figure 6D: Monoblepharomycota positively correlated with OM (*P* < 0.05). Basidiobolomycota was strongly positively correlated with TP (*P* ≤ 0.001) and negatively correlated with OM (*P* < 0.05). Mucoromycota displayed a significant positive correlation with pH (*P* < 0.01), negative correlations with TN and TP (*P* < 0.05), and a significant negative correlation with AK (*P* < 0.01). Glomeromycota showed positive correlations with NH_4_^+^-N and pH (*P* < 0.05), a negative correlation with AP (*P* < 0.05), and a significant negative correlation with AN (*P* < 0.01). Chytridiomycota positively correlated with AN (*P* < 0.05) and showed a significant positive correlation with OM (*P* < 0.01). Rozellomycota was strongly positively correlated with AK (*P* ≤ 0.001), significantly positively correlated with AP and TN (*P* < 0.01), positively correlated with AN (*P* < 0.05), and showed a significant negative correlation with NH_4_^+^-N (*P* < 0.01) and negative correlation with pH (*P* < 0.05). Basidiomycota positively correlated with pH (*P* < 0.05) and negatively correlated with AN and AP (*P* < 0.05). Ascomycota demonstrated a significant positive correlation with AN (*P* < 0.01) and positively correlated with OM (*P* < 0.05). These findings indicate that different composite modes have distinct impacts on the relationship between the dominant microbial species in *B. striata* roots and environmental factors. *B. striata* under different composite modes affect related physicochemical properties in the soil, subsequently influencing the composition and distribution of the microbial community.

## 4 Discussion

### 4.1 Relationship between bacterial communities and environmental factors

Interplanting strategies effectively harness available arable land, moisture, nutrients, and thermophotonic resources, bolstering the crop diversity index. By leveraging interspecific competition and complementarity, these techniques enhance agricultural yield and efficiency (Dapaah et al., 2003). Concurrently, they amplify aboveground and belowground interspecific interactions in fields, augmenting, and modulating soil biodiversity. This in turn improves the microecological quality of the soil and alleviates obstacles associated with continuous cropping (Li et al., 2023). Existing studies underscore the pivotal role soil OM, nitrogen, and phosphorus play in uplifting crop yield and quality. The soil quality is predominantly influenced by soil OM affecting soil pH, moisture retention, and the concentration of soil nutrients, among other pedological traits (Ruthes et al., 2023). Intercropping can significantly shape the structure and diversity of soil microbial communities, elevating the count of beneficial microorganisms while suppressing pathogens. Such practices are instrumental in refining the soil environment and fortifying plant disease resistance (Li et al., 2014). Our findings reveal that, in comparison to the monocropping system, the silvo-medicinal intercropping management has a notable impact on soil nutrients, including OM, TN, TP, TK, AN, AP, AK, NH_4_^+^-N, and NO_3_^–^-N, as well as on pH levels (Table 1). Specifically, in the TB system, there was an increase in OM, TN, AN, AP, AK, and NO_3_^–^-N levels, with a concurrent reduction in TP, TK, NH_4_^+^-N levels, and pH values. In contrast, the PB system elevated the levels of TN, TP, AN, AP, and AK, but reduced OM, TK, and NO_3_^–^-N levels, along with pH values. The LB system resulted in heightened levels of TN, TK, AP, AK, and NO_3_^–^-N, but exhibited a decrease in NH_4_^+^-N levels and pH values. Generally, in the intercropping systems, the levels of TN, AK, AP, and AN consistently surged, while NH_4_^+^-N levels and pH values markedly dwindled. This could be attributed to the significant variations in the canopy type, litterfall, and root exudates of *B. striata* in different forest settings, thus triggering alterations in soil nutrients (Zhang Y. et al., 2021; Li X. et al., 2022). Notably, in a majority of ecological systems, over 90% of the nitrogen and phosphorus required by plants from the soil are sourced from the decomposition of litterfall (Lucas-Borja et al., 2019), which corroborates the findings of Zhang X. et al. (2020).

Although numerous studies have explored the effects of tree leaf litter on soil microbiota, research investigating the structure and functional response of soil microbes beneath fruit trees to the decomposition of *B. striata* leaf litter remains uncharted territory. The decomposition of leaves and fallen fruits under fruit trees plays a pivotal role in shaping the soil nutrient profile and microbial community structure. Moreover, distinct soil characteristics can induce shifts in soil microbial assemblies (Shi et al., 2016). In the present study, the abundance of *Actinobacteriota* and *Chloroflexi* displayed a significant positive correlation with NH_4_^+^-N and pH, but a negative correlation with AN, AK, and AP. *Bacteroidota* showed a positive association with AK, AP, and TN, yet a negative relationship with NH_4_^+^-N and pH (Figure 6C). Meanwhile, Myxococcota negatively correlated with OM. Our survey identified Proteobacteria, Acidobacteriota, Firmicutes, Bacteroidota, Actinobacteria, Chloroflexi, and Actinobacteriota as dominant bacterial phyla in both intercropping and monocropping systems. These are common bacterial taxa found in soils, aligning with earlier research findings (Fierer and Jackson, 2006; Janssen, 2006). Among these, the soil bacterium Proteobacteria emerged as the predominant phylum across all intercropping patterns and is recognized as a primary functional bacterium responsible for decomposition and transformation. This observation is congruent with previously reported findings (Mander et al., 2012; Huang et al., 2016).In the context of our findings, the bacterial phyla Acidobacteriota, Firmicutes, Chloroflexi, Verrucomicrobiota, Gemmatimonadetes, and Myxococcota exhibited significantly higher abundances in LB, TB, and PB intercropping systems compared to monocropping (Figure 4). Within the PB system, the relative abundance of Bacteroidota was lower than in C, whereas in the TB and LB systems, the relative abundance of Actinobacteria was lower than in C (Supplementary Table 2).

Though Acidobacteriota is ubiquitous across various soil types, it exemplifies soils under acidic conditions and showcases diverse metabolic capabilities, particularly linked to OM decomposition (Jones et al., 2009; Kielak et al., 2016). This explains the decrease in soil pH in the rhizosphere of *B. striata* post-fruit tree intercropping. Firmicutes bacteria possess the capacity to ferment complex polysaccharides and other organic substances and play an instrumental role in beneficial plant-root interactions (Yutin and Galperin, 2013). This capability might be related to the synthesis of *B. striata* polysaccharides. Chloroflexi, on the other hand, aids in the decomposition of OM in the soil, contributing to organic carbon cycling and nitrogen cycling, whilst interacting with other soil microbial communities, thus impacting soil structure and stability (Hug et al., 2013; Kielak et al., 2016). A salient feature of Myxococcota, one of the few known predatory bacteria, is its ability to prey on other bacteria or organic substances. These bacteria can form unique multicellular structures, enabling them to withstand unfavorable environmental conditions over extended periods. Furthermore, their presence is instrumental in maintaining soil structure and enhancing its mechanical stability (Shimkets, 1990; Reichenbach, 1999; Berleman and Kirby, 2009; Weissman and Müller, 2010). When comparing the relative abundance of dominant bacterial genera in different soil conditions, we observed that after intercropping, the abundance of *Pseudomonas*, *Sphingomonas*, and *Pseudarthrobacter* declined compared to monocultures. These bacteria are known to decompose OM and facilitate ecological restoration in soils (Stolz, 2009). In contrast, the abundance of *Bryobacter*, *Bradyrhizobium*, *Gemmatimonas*, *Acidibacter*, *Haliangium*, and *Reyranella* increased relative to monocultures. These bacterial genera play critical roles in decomposing OM, fixing atmospheric nitrogen, and enhancing soil fertility (Barns et al., 2007; Alippi et al., 2012). The findings indicate that soil conditions improved following the intercropping of *B. striata* with fruit trees, resulting in the suppression of *Pseudomonas*, *Sphingomonas*, and *Pseudarthrobacter* growth, and the promotion of *Bryobacter*, *Bradyrhizobium*, *Gemmatimonas*, *Acidibacter*, *Haliangium*, and *Reyranella* growth. Additionally, in this study, the genera labeled as “Others” and “Unidentified” accounted for over 50% and 6.8%, respectively, in both systems. This suggests that there remains a substantial number of unknown or rare bacteria in the soil under *B. striata* that require further identification and research.

### 4.2 Relationship between fungal communities and environmental factors

Previous studies have suggested that orchard intercropping can significantly increase microbial community diversity (Dahlstrom et al., 2020; Ding et al., 2021; Zhu et al., 2022). In contrast, other research has indicated that while intercropping reduces the number of soil microbes, it does not compromise their richness (Zhong et al., 2018). Such findings underscore the notion that the impact of intercropping on soil microbial diversity may vary depending on the specific plants involved in the intercrop. In our study, the indices related to fungi were generally higher in monocultures than in intercropping systems. This suggests that, over a certain period, the intercropping of fruit trees with *B. striata* leads to a decline in both the quantity and diversity of fungi. The significantly reduced fungal richness in PB and LB compared to monocultures could be attributed to the presence of acidic substances, such as malic and citric acids, in fallen apples and pears. As these fruits decompose in the soil, the resulting organic acids may acidify the soil (Gallinger et al., 2021), potentially contributing to the observed reduction in fungal numbers. Given that our sampling coincided with the period of apple and pear drop, further observations are needed to discern the impact of soil pH and microbial abundance changes.

Significantly, TB enhanced the Chao1 index of *B. striata* rhizosphere soil, suggesting that TB can effectively boost the fungal community diversity in *B. striata* root soil, playing a crucial role in enhancing the soil’s ecological environment and microbial diversity. Additionally, dominant microbial communities in both intercropped and monocultured soils included beneficial microbes for plants, such as Basidiomycota, Ascomycota, Chytridiomycota, and Glomeromycota (Sommermann et al., 2018; Li Y. et al., 2022; Sun P. et al., 2022; Wang et al., 2022). These microbes facilitate OM recycling, decompose organic materials, and establish mycorrhizal symbiotic relationships with plants. Notably, in this study, Ascomycota emerged as the predominant fungal phylum capable of decomposing soil OM, promoting the cycling of key nutrients and enhancing soil fertility (Brakhage, 2013). Their abundance showed a significant positive correlation with AN and OM (Figure 6D).

Rozellomycota is understood to be a group of fungi predominantly characterized by their parasitic and symbiotic lifestyles. They potentially play pivotal roles in OM decomposition, soil structure formation, and interactions with other soil biota, such as establishing symbiotic relationships with plants (Jones et al., 2011). Their abundance exhibited a significant positive correlation with AK, AN, AP, and TN while showing a pronounced negative correlation with NH_4_^+^-N and pH (Figure 6D). *Mucoromycota*, on the other hand, can serve as biological pesticides, combating soil-borne pests and pathogenic microbes. They form endomycorrhizal symbiotic relationships with plants and participate in OM decomposition (Spatafora et al., 2016; Orchard et al., 2017). Their abundance is significantly positively correlated with pH but negatively associated with AK, TN, and TP (Figure 6D). *B. striata* is a shade-loving plant that is averse to strong sunlight. The results suggest that intercropping generates favorable impacts on soil nutrient levels and microbial community composition. Moreover, microbes appear to play an integral role in nutrient cycling. This insight aids in further evaluating the implications of intercropping fruit trees with *B. striata* on the physicochemical properties of soil.

## 5 Conclusion

The mixed planting of fruit trees and *B. striata* significantly elevates the levels of AN, AP, and AK in the soil, concurrently reducing NH_4_^+^-N content and pH values. Additionally, this integrated cultivation strategy modifies the soil microbial community, notably increasing the relative abundance of Acidobacteriota and Firmicutes phyla. A marked correlation exists between soil microbes and soil physicochemical properties, with NH_4_^+^-N and pH demonstrating significant relationships with both bacterial and fungal communities. Beyond the enhancement of the bacterial community structure within the soil, there is a noted rise in the relative abundance of beneficial bacterial and fungal groups linked primarily to disease prevention and nutrient cycling. In essence, the integration of *B. striata* with fruit trees can considerably augment soil nutrient levels, and refine the soil bacterial microbiome in terms of composition and diversity, albeit leading to soil acidification and depletion of NH_4_^+^-N. For such agroforestry systems, adopting measures like the application of controlled or slow-release nitrogen fertilizers and the addition of lime or wood ash can ameliorate soil pH. Comprehensive long-term analyses of these intercropping systems are requisite, with a focus on identifying the most advantageous planting patterns for farmers. Such findings are expected to provide theoretical underpinnings for ensuing agricultural practices and research.

## Data availability statement

The original contributions presented in the study are publicly available. This data can be found here: https://www.ncbi.nlm.nih.gov/genbank/, PRJNA1021261.

## Author contributions

QX: Conceptualization, Formal analysis, Investigation, Software, Writing – original draft. HX: Formal analysis, Investigation, Writing – original draft. RW: Data curation, Formal analysis, Investigation, Writing – original draft. LW: Data curation, Formal analysis, Investigation, Writing – original draft. YY: Formal analysis, Software, Writing – original draft. HZ: Resources, Supervision, Validation, Writing – review & editing. BS: Resources, Validation, Writing – review and editing.

## Footnotes

1
http://ccb.jhu.edu/software/FLASH/

2
http://qiime.org/scripts/split_libraries_fastq.html

3
https://github.com/torognes/vsearch/

4
https://cloud.oebiotech.com/

5
https://www.cloudtutu.com
