# Supplementary material for: Integrated management of fruit trees and Bletilla striata: implications for soil nutrient profiles and microbial community structures
Source: Front Microbiol. 2024 Mar 6;15:1307677. doi: 10.3389/fmicb.2024.1307677 (PMC10951077; doi:10.3389/fmicb.2024.1307677)
Supplement: Supplementary file 1 [file Table_1.DOC]

Supplementary tables

Table S1. ANOSIM and permutational MANOVA of different treatments effects on Soil microbial diversity

| **Treatments** | **Bacteria** | | | | **Fungi** | | | |
| --- | --- | --- | --- | --- | --- | --- | --- | --- |
|  | **ANOSIM** | | **ADONIS** | | **ANOSIM** | | **ADONIS** | |
|  | **R** | ***P*** | **R^2^** | ***P*** | **R** | ***P*** | **R^2^** | ***P*** |
| PB-vs-TB-vs-LB-vs-C | 0.8117 | 0.001 | 0.7877 | 0.001 | 0.4907 | 0.001 | 0.3867 | 0.146 |

LB: Bletilla *striata* intercropped with Pear orchard;PB: *Bletilla striata* intercropped with Apple orchard;TB: *Bletilla striata* intercropped with Peach orchard;C: Monoculture of *Bletilla striata*.

**Table S2.** Relative abundance of bacteria taxa from intercropping soils

| **Level** | **Phylum** | **PB** | **TB** | **LB** | **C** | ***P*-value** | **Significant** |
| --- | --- | --- | --- | --- | --- | --- | --- |
| Phylum | *Myxococcota* | 0.02±0.00ab | 0.03±0.01a | 0.03±0.01a | 0.02±0.00b | 0.030 | * |
|  | *Gemmatimonadetes* | 0.05±0.02a | 0.03±0.01ab | 0.05±0.02a | 0.01±0.00b | 0.018 | * |
|  | *Verrucomicrobiota* | 0.04±0.01a | 0.05±0.02a | 0.04±0.02a | 0.02±0.01a | 0.254 |  |
|  | *Actinobacteriota* | 0.07±0.02a | 0.03±0.00b | 0.03±0.00b | 0.03±0.00b | 0.006 | ** |
|  | *Chloroflexi* | 0.12±0.04a | 0.04±0.01b | 0.02±0.00b | 0.02±0.00b | 0.001 | ** |
|  | *Actinobacteria* | 0.07±0.04a | 0.05±0.01a | 0.05±0.01a | 0.07±0.03a | 0.285 |  |
|  | *Bacteroidota* | 0.04±0.01c | 0.11±0.01a | 0.10±0.03ab | 0.06±0.02bc | 0.007 | ** |
|  | *Firmicutes* | 0.03±0.03ab | 0.16±0.10ab | 0.19±0.12a | 0.02±0.02b | 0.060 |  |
|  | *Acidobacteriota* | 0.17±0.05a | 0.16±0.02a | 0.12±0.02ab | 0.06±0.03b | 0.015 | * |
|  | *Proteobacteria* | 0.28±0.02b | 0.28±0.05b | 0.32±0.08b | 0.66±0.05a | 0 | ** |
| Genus | *Reyranella* | 0.01±0.00a | 0.01±0.00ab | ＜0.01bc | ＜0.01c | 0.017 | * |
|  | *Haliangium* | ＜0.01bc | 0.01±0.00a | 0.01±0.00ab | ＜0.01c | 0.006 | ** |
|  | *unidentified_Clostridiaceae* | ＜0.01b | 0.01±0.01ab | 0.02±0.02a | ＜0.01b | 0.073 |  |
|  | *Acidibacter* | 0.01±0.00a | 0.01±0.00a | 0.01±0.00a | 0.01±0.00a | 0.811 |  |
|  | *Gemmatimonas* | 0.02±0.01a | 0.02±0.00a | 0.02±0.01a | 0.02±0.00a | 0.268 |  |
|  | *Bradyrhizobium* | 0.02±0.01a | 0.01±0.00a | 0.02±0.01a | 0.01±0.00a | 0.219 |  |
|  | *Bryobacter* | 0.03±0.02a | 0.02±0.01a | 0.01±0.00a | 0.01±0.00a | 0.126 |  |
|  | *Pseudarthrobacter* | 0.01±0.00a | 0.01±0.00a | 0.02±0.01a | 0.03±0.03a | 0.201 |  |
|  | *Sphingomonas* | 0.02±0.01a | 0.02±0.00a | 0.04±0.02a | 0.05±0.06a | 0.503 |  |
|  | *Pseudomonas* | 0.01±0.02b | 0.01±0.00b | ＜0.01b | 0.37±0.14a | 0 | ** |

The top 10 are shown. Values are presented as mean ± standard error (n = 3). Different superscript letters indicate statistically significant differences (P < 0.05) between different treatments as determined by Tukey’s test. *, indicates a significant difference at the P < 0.05 level; **, indicates a significant difference at the P < 0.01 level; ***, indicates a significant difference at the P < 0.001 level. LB: *Bletilla striata* intercropped with Pear orchard; PB: *Bletilla striata* intercropped with Apple orchard; TB: *Bletilla striata* intercropped with Peach orchard; C: Monoculture of *Bletilla striata*.

Table S3. Relative abundance of fungi taxa from intercropping soils

| **Level** | **Phylum** | **PB** | **TB** | **LB** | **C** | ***P*-value** | **Significant** |
| --- | --- | --- | --- | --- | --- | --- | --- |
| Phylum | *Ascomycota* | 0.36±0.10b | 0.39±0.03b | 0.48±0.06ab | 0.56±0.04a | 0.019 | * |
|  | *Basidiomycota* | 0.21±0.12a | 0.14±0.00a | 0.11±0.02a | 0.09±0.03a | 0.190 |  |
|  | *Mortierellomycota* | 0.04±0.03a | 0.04±0.00a | 0.07±0.04a | 0.05±0.02a | 0.478 |  |
|  | *Chytridiomycota* | ＜0.01b | ＜0.01ab | ＜0.01b | 0.01±0.01a | 0.040 | * |
|  | *Rozellomycota* | ＜0.01b | 0.01±0.00a | 0.01±0.00a | 0.01±0.00a | 0.014 | * |
|  | *Glomeromycota* | ＜0.01a | ＜0.01ab | ＜0.01b | ＜0.01b | 0.019 | * |
|  | *Mucoromycota* | ＜0.01a | ＜0.01b | ＜0.01c | ＜0.01ab | 0.001 | ** |
|  | *Basidiobolomycota* | ＜0.01b | ＜0.01b | ＜0.01a | ＜0.01b | 0.013 | * |
|  | *Zoopagomycota* | ＜0.01a | ＜0.01a | ＜0.01a | ＜0.01a | 0.498 |  |
|  | *Monoblepharomycota* | ＜0.01a | ＜0.01a | ＜0.01a | ＜0.01a | 0.171 |  |
| Genus | *Serendipita* | 0.05±0.00a | 0.08±0.00a | 0.05±0.00a | 0.08±0.00a | 0.425 |  |
|  | *Fusarium* | 0.05±0.04b | 0.02±0.00b | 0.09±0.01a | 0.02±0.00b | 0.010 | * |
|  | *Hygrocybe* | 0.04±0.06a | ＜0.01a | ＜0.01a | ＜0.01a | 0.438 |  |
|  | *Cladorrhinum* | ＜0.01b | ＜0.01b | 0.06±0.04a | ＜0.01b | 0.039 | * |
|  | *Chaetomium* | 0.02±0.02a | 0.01±0.00a | 0.05±0.04a | 0.01±0.00a | 0.178 |  |
|  | *Neurospora* | ＜0.01b | ＜0.01b | 0.05±0.04a | ＜0.01b | 0.038 | * |
|  | *unidentified* | 0.02±0.02a | 0.01±0.00a | 0.02±0.02a | 0.01±0.00a | 0.195 |  |
|  | *Cladosporium* | ＜0.01c | 0.01±0.00b | ＜0.01c | 0.05±0.01a | 0 | ** |
|  | *Thysanorea* | ＜0.01b | ＜0.01b | ＜0.01b | 0.05±0.01a | 0 | ** |
|  | *f__Nectriaceae;g__unidentified* | 0.02±0.01b | 0.04±0.01a | 0.02±0.00b | 0.01±0.01b | 0.001 | ** |

The top 10 are shown. Values are presented as mean ± standard error (n = 3). Different superscript letters indicate statistically significant differences (P < 0.05) between different treatments as determined by Tukey’s test. *, indicates a significant difference at the P < 0.05 level; **, indicates a significant difference at the P < 0.01 level; ***, indicates a significant difference at the P < 0.001 level. LB: *Bletilla striata* intercropped with Pear orchard;PB: *Bletilla striata* intercropped with Apple orchard;TB: *Bletilla striata* intercropped with Peach orchard;C: Monoculture of *Bletilla striata*.

Table S4. Results of ANOVA like permutation test on the relationship between soil properties and microbial communities

| **Variables** | **Bacteria** | | **Fungi** | |
| --- | --- | --- | --- | --- |
|  | **R^2^** | ***P*-values** | **R^2^** | ***P*-values** |
| OM | 0.85 | 0.003 | 0.76 | 0.003 |
| TN | 0.77 | 0.003 | 0.13 | 0.511 |
| TP | 0.28 | 0.236 | 0.53 | 0.028 |
| TK | 0.14 | 0.500 | 0.18 | 0.423 |
| AN | 0.80 | 0.001 | 0.77 | 0.001 |
| AP | 0.83 | 0.006 | 0.54 | 0.037 |
| AK | 0.83 | 0.004 | 0.47 | 0.07 |
| NH_4_^+^-N | 0.81 | 0.006 | 0.45 | 0.075 |
| NO_3_^-^-N | 0.20 | 0.347 | 0.07 | 0.721 |
| pH | 0.83 | 0.002 | 0.57 | 0.027 |

OM，Organic Matter；TN， Total Nitrogen；TP，Total Phosphorus；TK，Total Potassium；AN，Available Nitrogen；AP，Available Phosphorus；AK，Available Potassium；NH_4_^+^-N，Ammonium Nitrogen；NO_3_^-^-N，Nitrate Nitrogen
