# Supplementary material for: Integrated management of fruit trees and Bletilla striata: implications for soil nutrient profiles and microbial community structures
Source: Front Microbiol. 2024 Mar 6;15:1307677. doi: 10.3389/fmicb.2024.1307677 (PMC10951077; doi:10.3389/fmicb.2024.1307677)
Supplement: Supplementary file 2 [file Data_Sheet_1.ZIP › Supplementary material/16s/20230110053345Agilent 5400╝∞▓Γ│ú╝√╬╩╠Γ╜Γ┤≡.pdf]

Agilent 5400检测常见问题解答

目 录

一、Agilent 5400检测和凝胶电泳检测的区别及优势 ———————— 3

二、报告里取消核酸检测结果的原因 ————————————————— 4

三、Agilent 5400检测相关产品文献 —————————————————— 5

四、Agilent 5400结果解读 ——————————————————————— 6

一、Agilent 5400检测和凝胶电泳检测的区别及优势

Agilent 5400全自动毛细管电泳系统是基于荧光检测技术和毛细管电泳技术发展而来的新型DNA/RNA片段分离仪器，DNA/RNA片段的分离是通过对毛细管阵列里填充的可导电的分离胶施加高压电场，来达到分离检测的目的。与传统琼脂糖凝胶电泳相比，具有更高的灵敏度和分辨率。Aglient 5400最大的特点是数据化的分析判定流程，与传统人工通过电泳条带判定相比，Aglient 5400对峰图进行smear区段的计算分析，判定结论较人工判定相比更稳定准确。

二、报告里取消核酸检测结果的原因

核酸检测的目的是为了给扩增环节参考确定扩增体系中的模板用量，我们的流程中采用梯度稀释扩增的方式进行扩增，流程中存在多个扩增体系可以涵盖样本的复杂情况，降低单个扩增体系的随机性，同时保证了扩增的合格率。扩增子产品建库的标准是扩增产物是否合格，所以报告中呈现的是扩增目的产物的扩增结果是否合格。

三、Agilent 5400结果解读

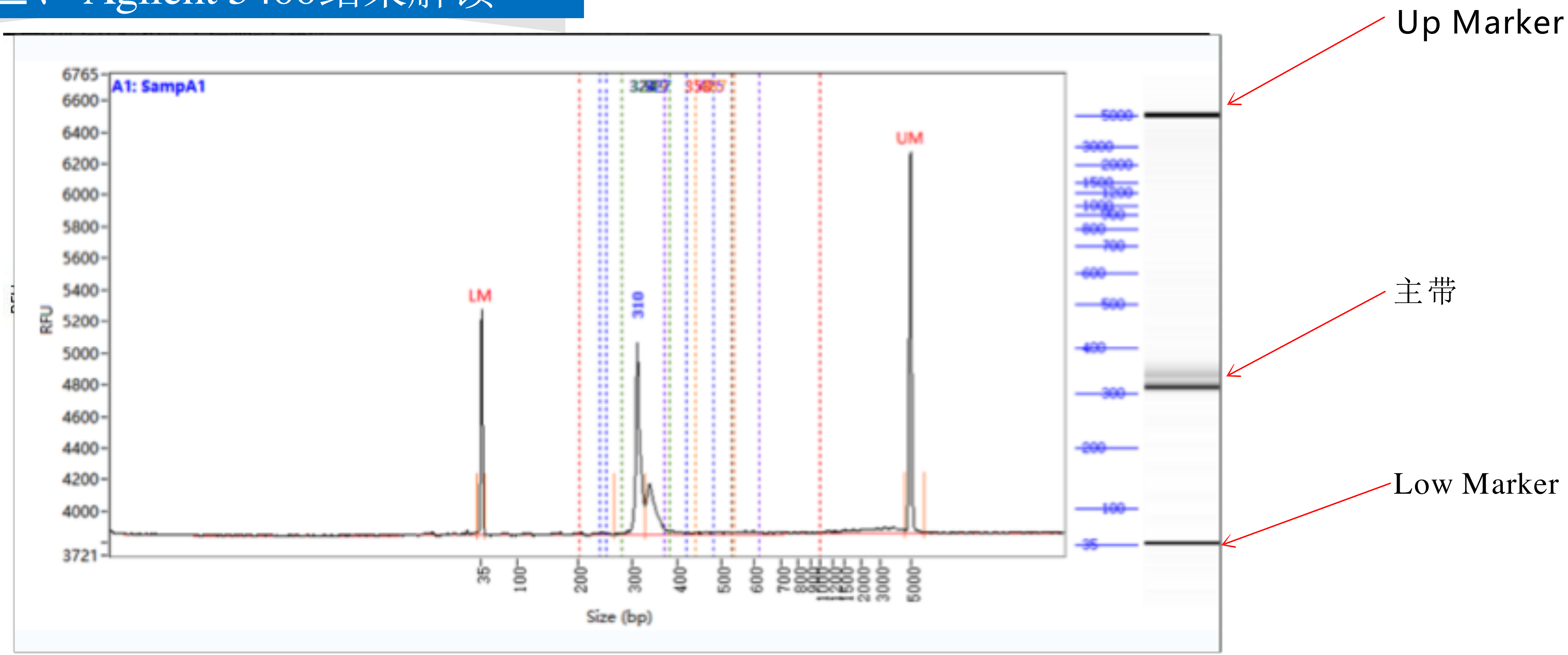

1、横坐标：size（bp）反应所在毛细管样品的片段分布情况；

2、纵坐标（RFU）值是指该样本在毛细管分离的过程中的实时荧光信号强度，RFU值越高、则样本浓度越高；

3、Low Marker/Up Marker：指随样本分析的参照物（非样本本身片段），用于标定样本的片段大小及浓度。

三、 Agilent 5400结果解读

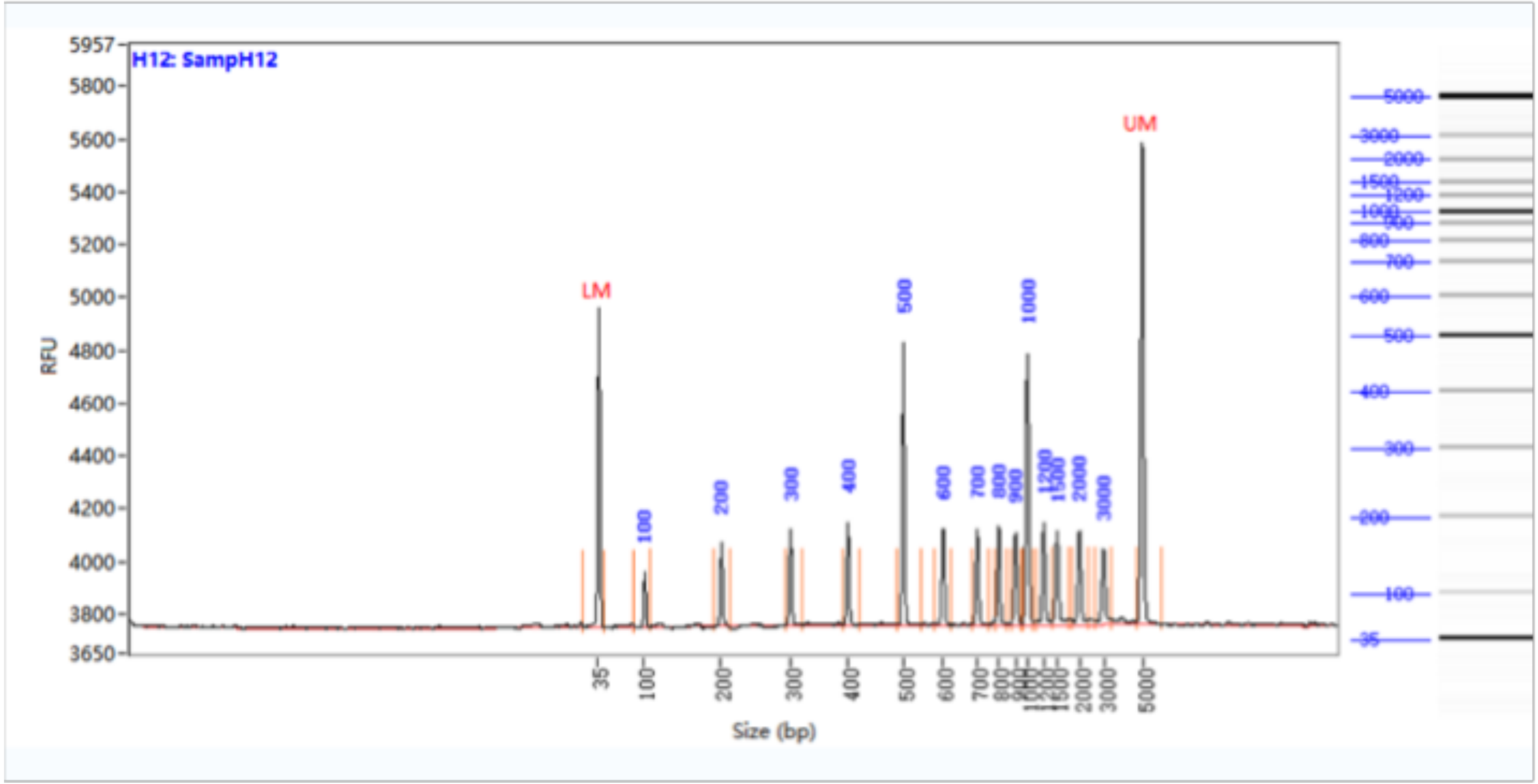

上图为Ladder size值大小标注

三、Agilent 5400结果解读

※ A（Pass） 16SV4

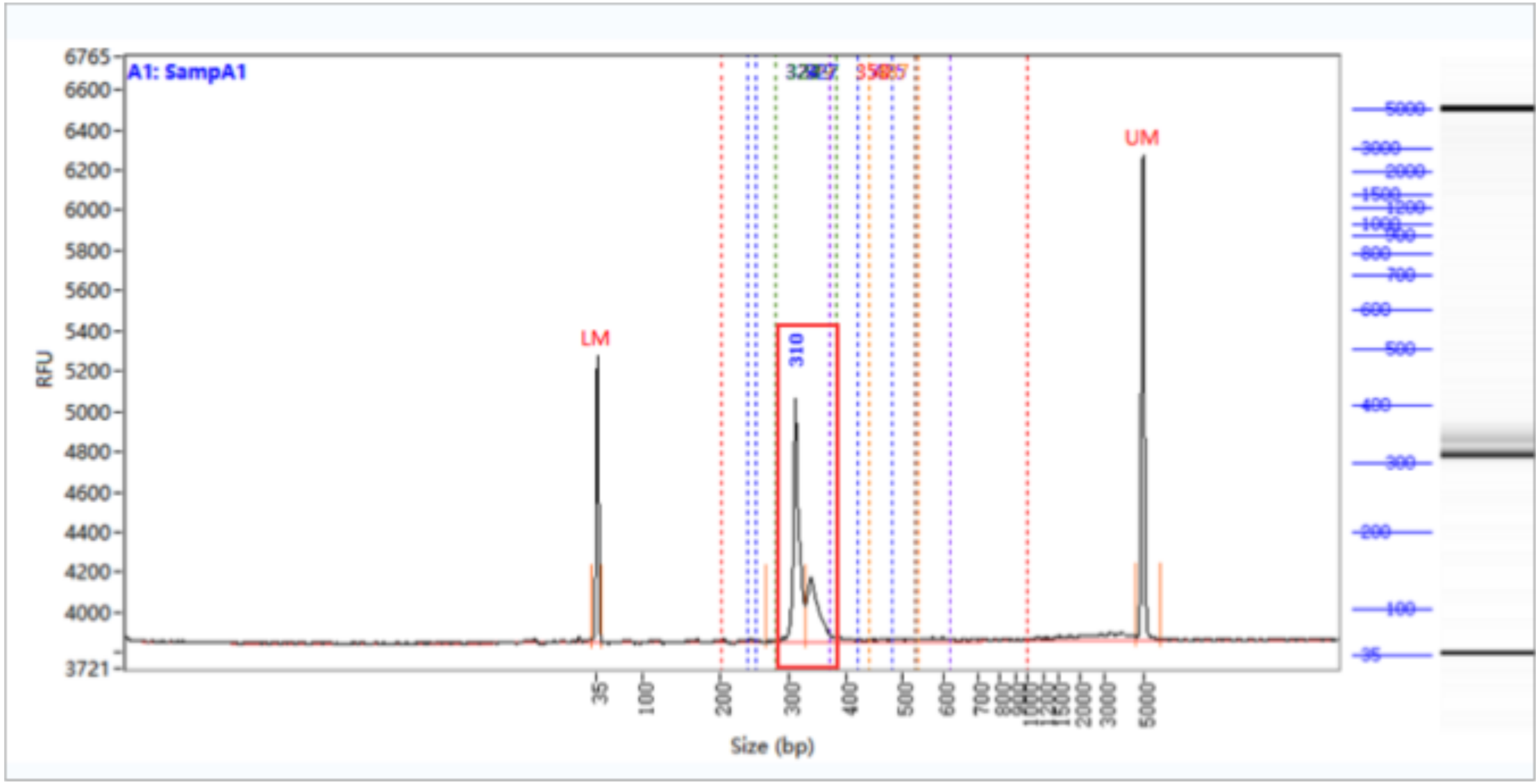

目标区域主带（红色框区域）清晰，浓度和总量均满足建库要求

三、 Agilent 5400结果解读

※ A (Pass) 16SV4

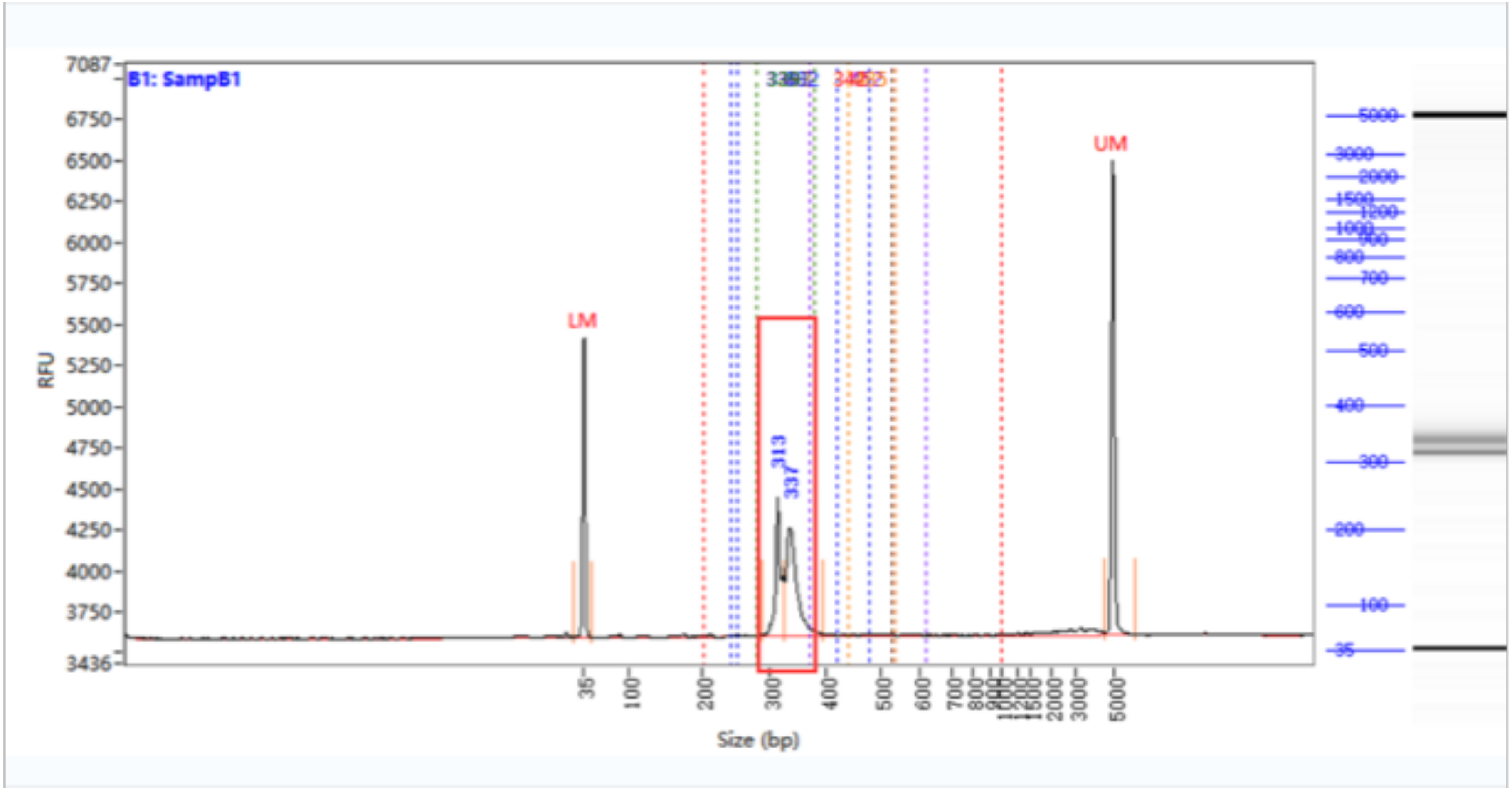

产物有两条明显主带且均在目标区域内，浓度和总量均满足建库要求

三、 Agilent 5400结果解读

※ C（Fail） 16SV4

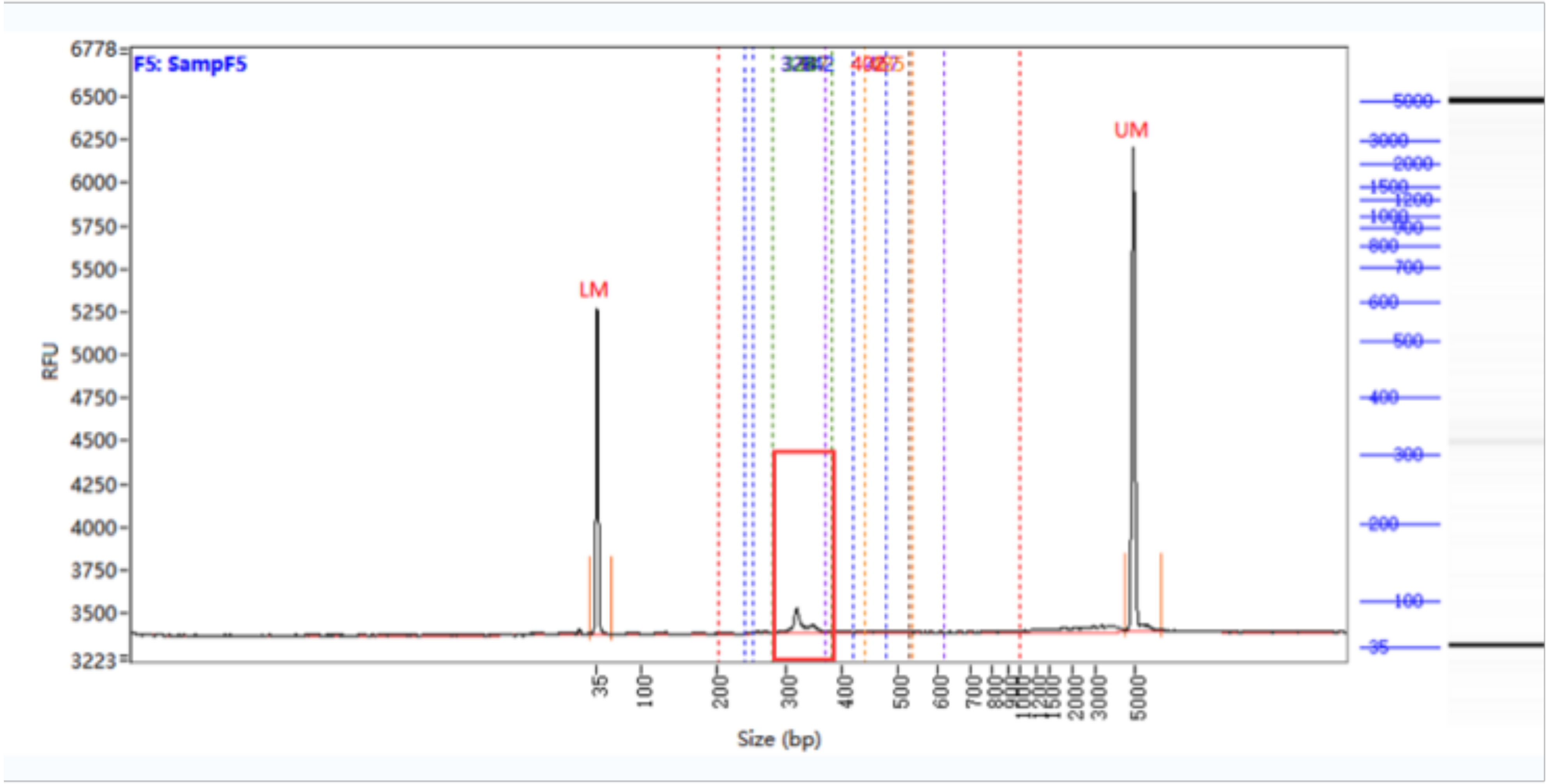

目标区域主带微弱，且浓度和总量均不满足建库要求

三、Agilent 5400结果解读

※ A（Pass） 16SV34

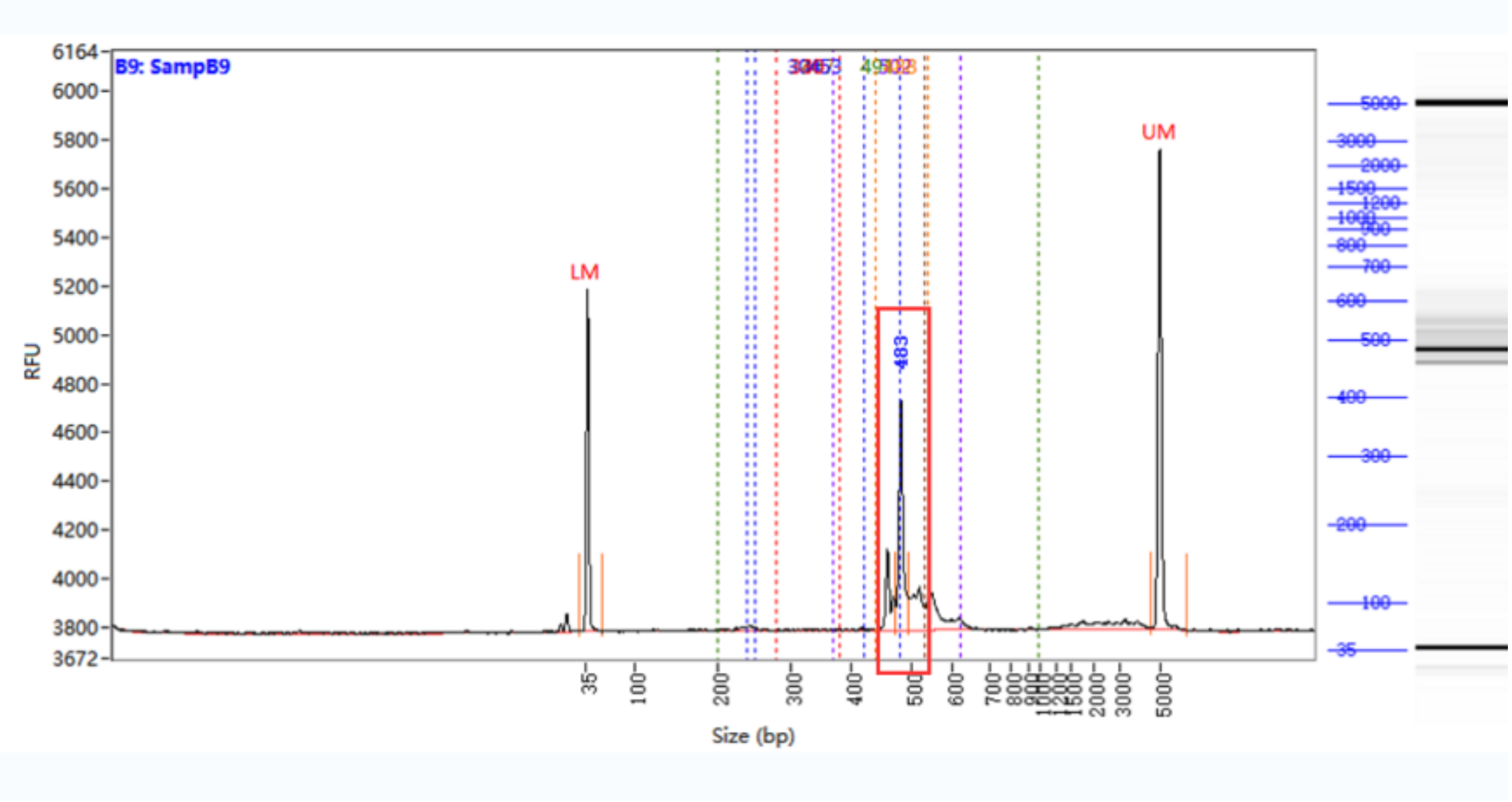

目标区域内存在多条主带，浓度和总量均满足建库要求

三、Agilent 5400结果解读

※ C（Fail） 16SV34

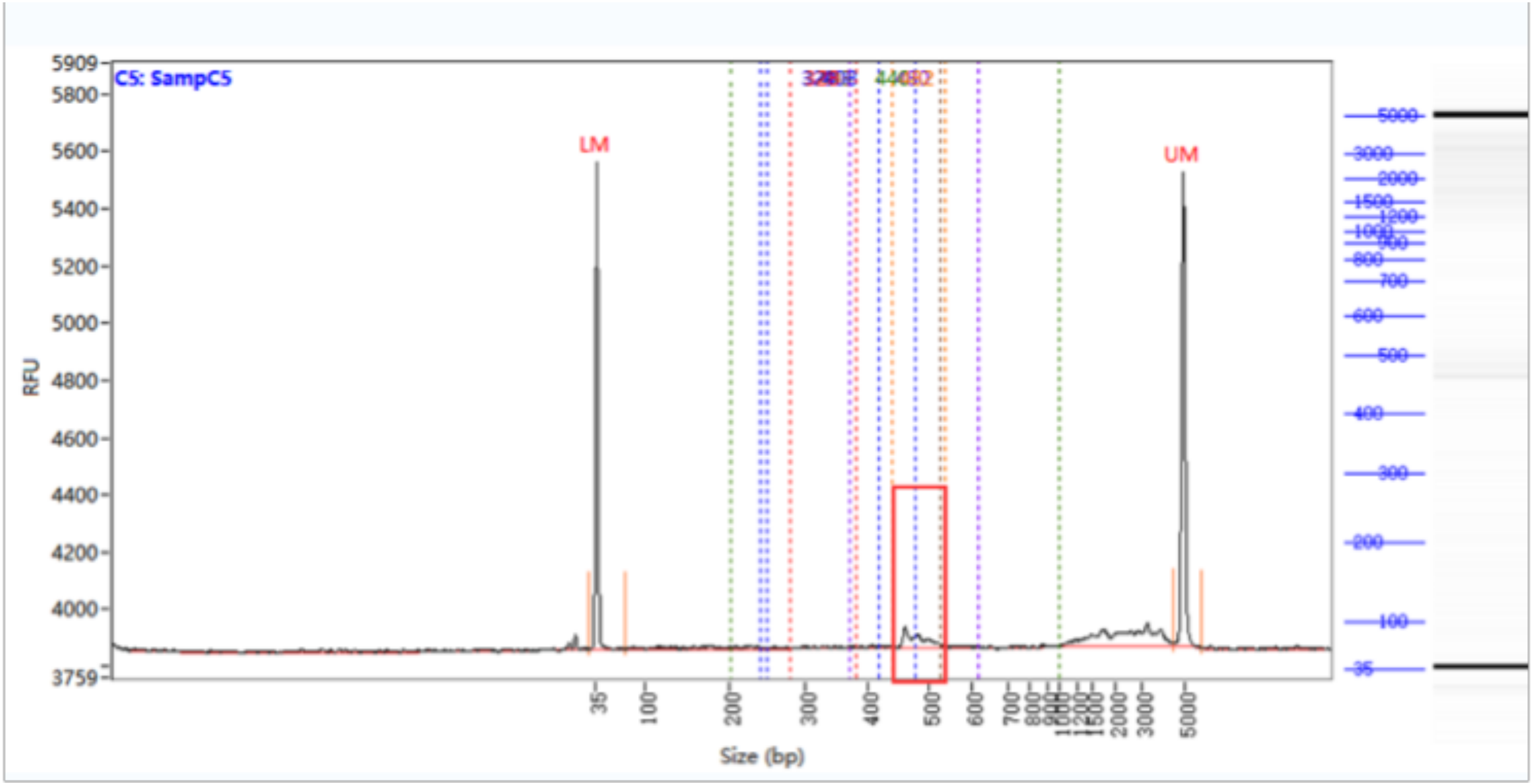

目标区域主带微弱，且浓度及总量均不满足建库要求

三、Agilent 5400结果解读

样本类型	质量指标	中文备注	英文备注	备注
DNA扩增产物	总量	扩增失败	Amplification failure	
