# Supplementary figures and images for: Integrated management of fruit trees and Bletilla striata: implications for soil nutrient profiles and microbial community structures
Source: Front Microbiol. 2024 Mar 6;15:1307677. doi: 10.3389/fmicb.2024.1307677 (PMC10951077; doi:10.3389/fmicb.2024.1307677)

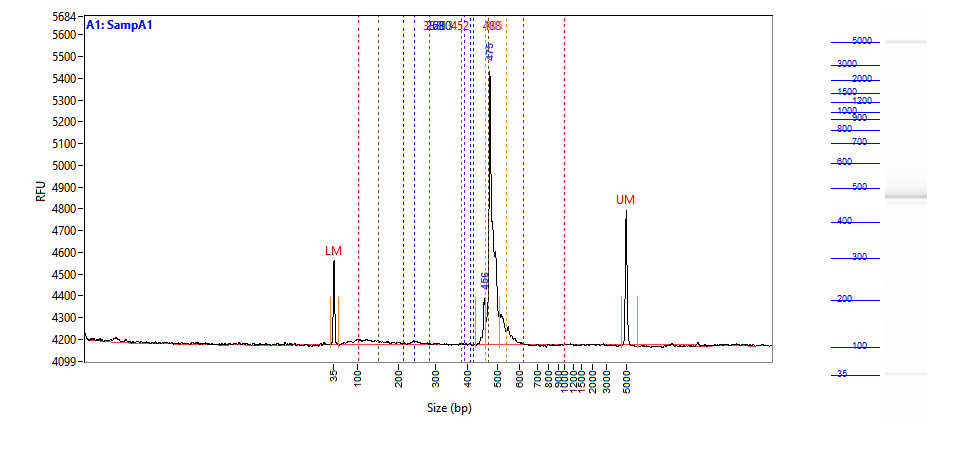

Supplement: Supplementary file 2 [file Data_Sheet_1.ZIP › Supplementary material/16s/16s-C1.BMP]

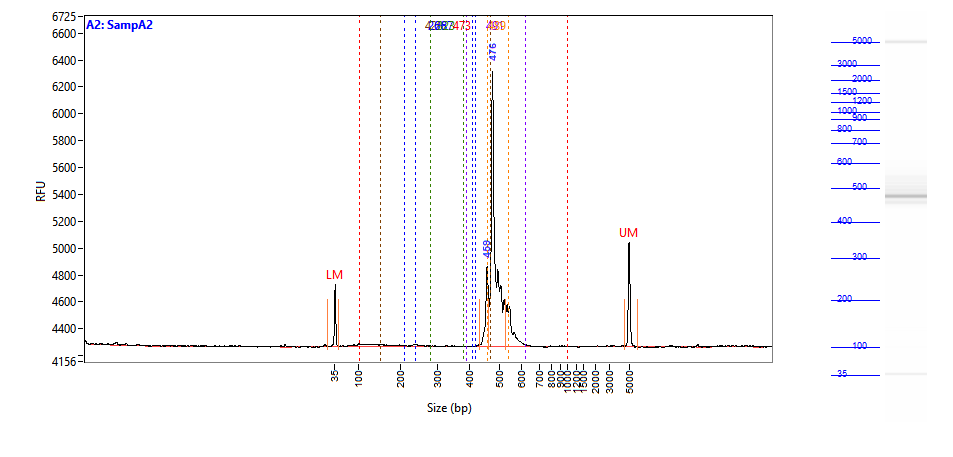

Supplement: Supplementary file 2 [file Data_Sheet_1.ZIP › Supplementary material/16s/16s-C2.BMP]

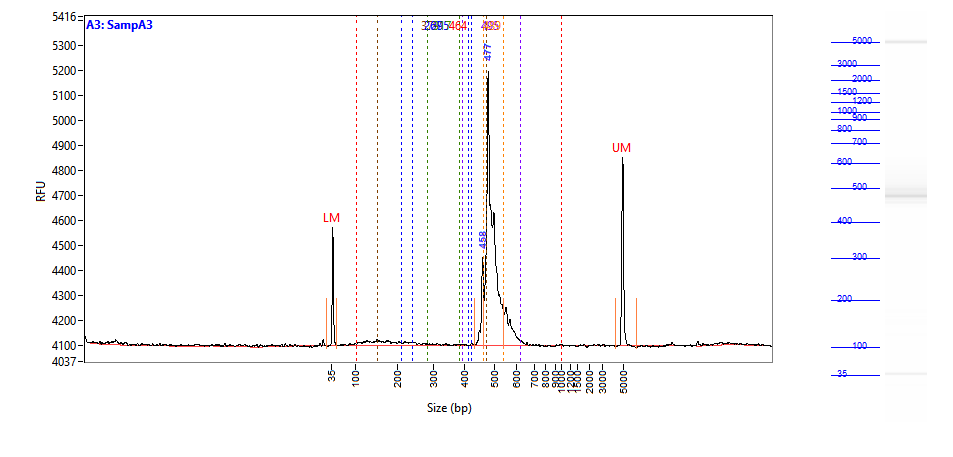

Supplement: Supplementary file 2 [file Data_Sheet_1.ZIP › Supplementary material/16s/16s-C3.BMP]

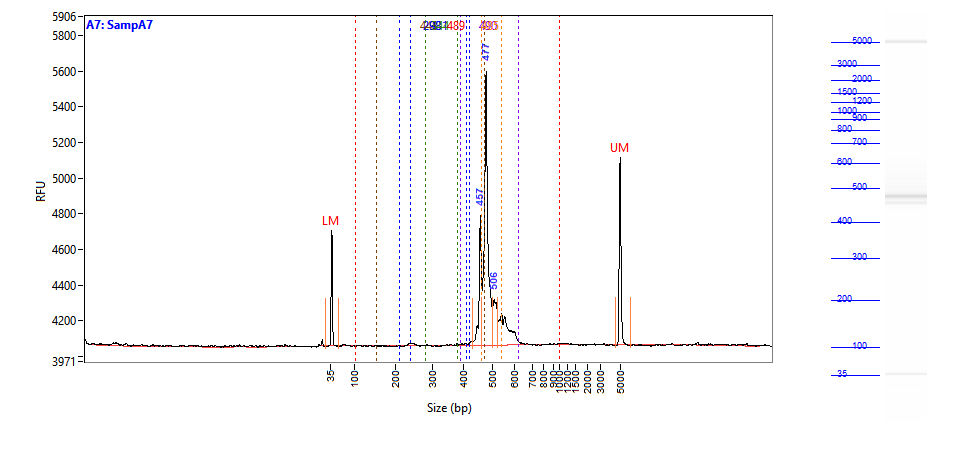

Supplement: Supplementary file 2 [file Data_Sheet_1.ZIP › Supplementary material/16s/16s-LB1.BMP]

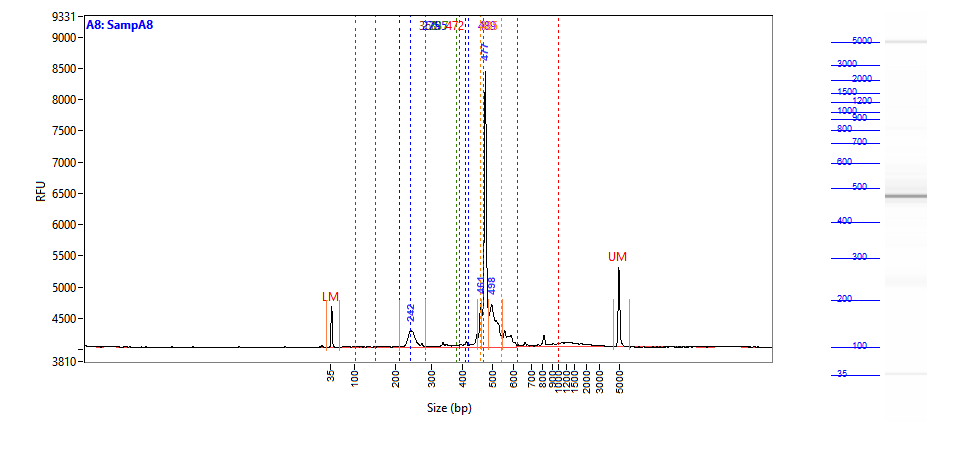

Supplement: Supplementary file 2 [file Data_Sheet_1.ZIP › Supplementary material/16s/16s-LB2.BMP]

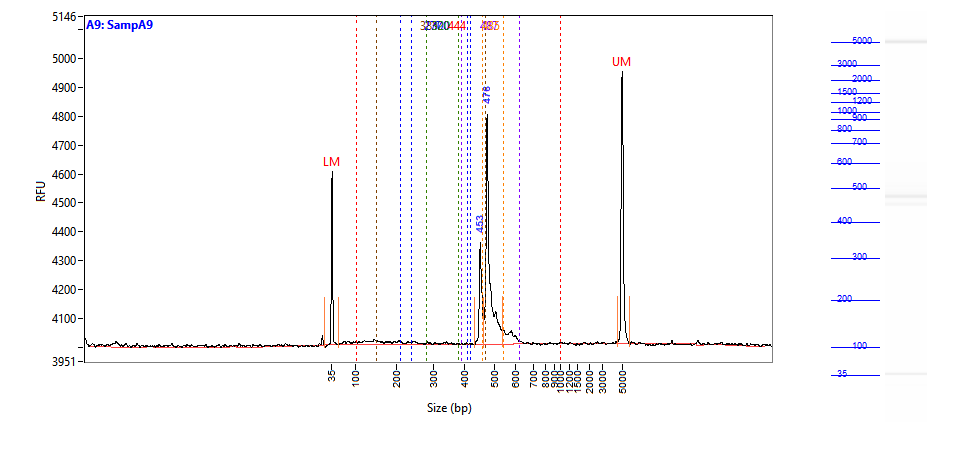

Supplement: Supplementary file 2 [file Data_Sheet_1.ZIP › Supplementary material/16s/16s-LB3.BMP]

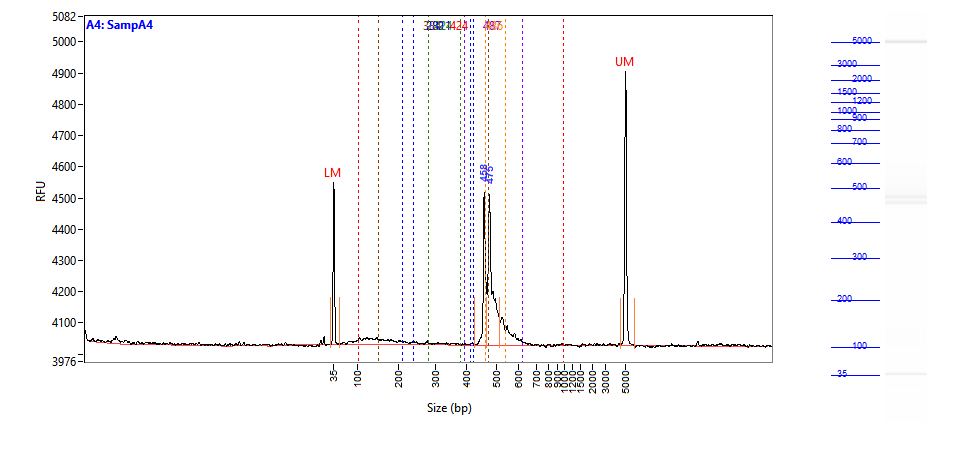

Supplement: Supplementary file 2 [file Data_Sheet_1.ZIP › Supplementary material/16s/16s-PB1.BMP]

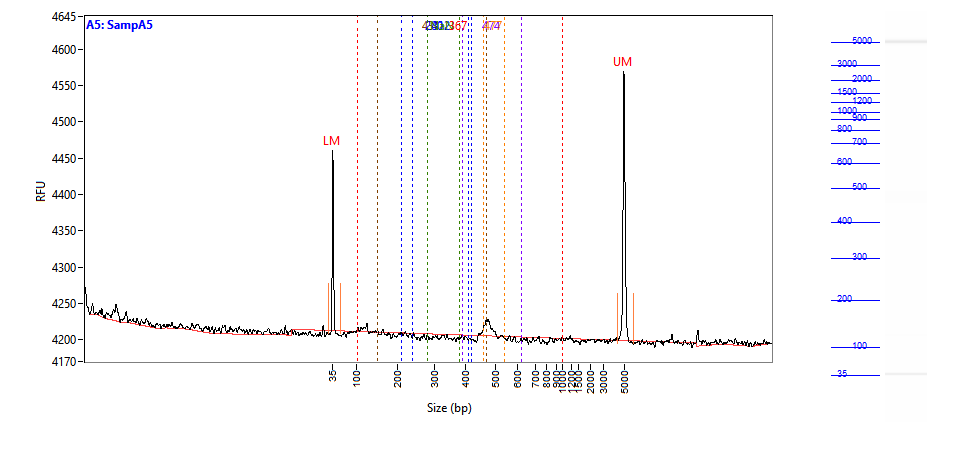

Supplement: Supplementary file 2 [file Data_Sheet_1.ZIP › Supplementary material/16s/16s-PB2.BMP]

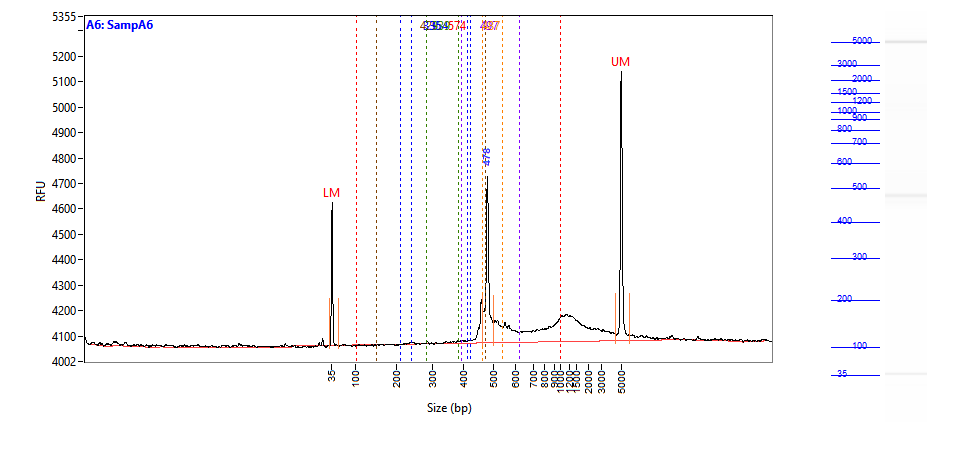

Supplement: Supplementary file 2 [file Data_Sheet_1.ZIP › Supplementary material/16s/16s-PB3.BMP]

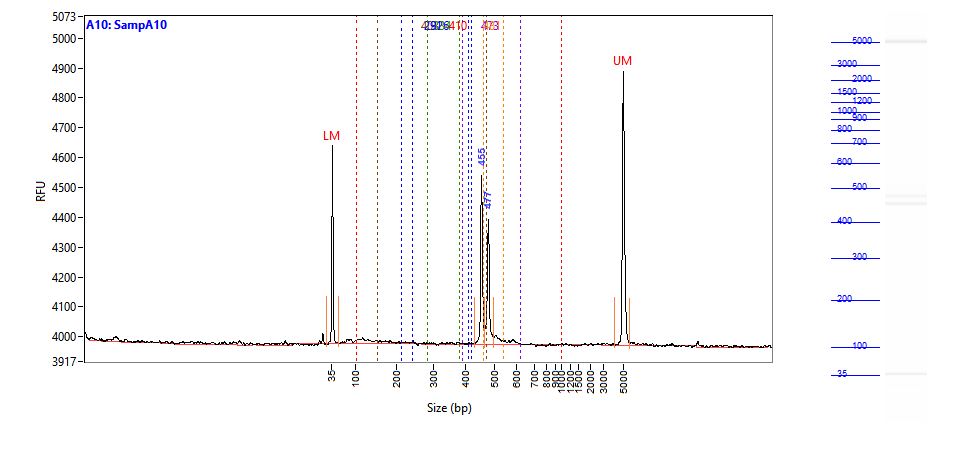

Supplement: Supplementary file 2 [file Data_Sheet_1.ZIP › Supplementary material/16s/16s-TB1.BMP]

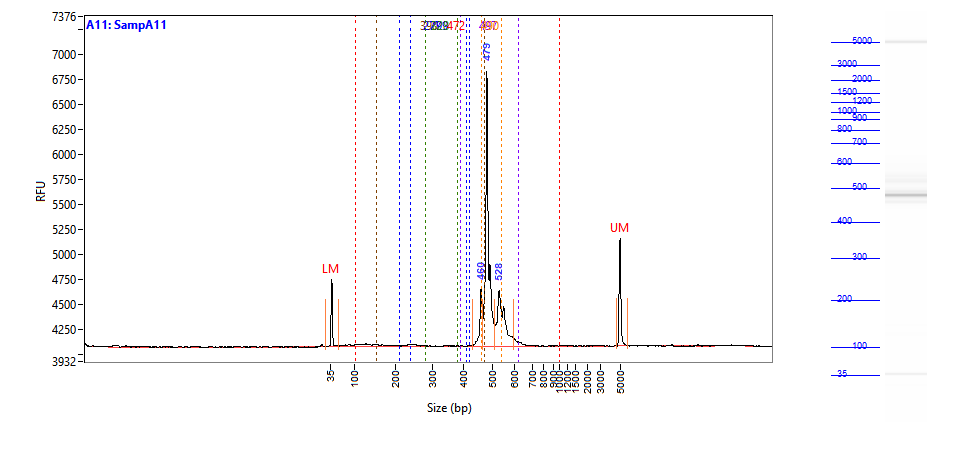

Supplement: Supplementary file 2 [file Data_Sheet_1.ZIP › Supplementary material/16s/16s-TB2.BMP]

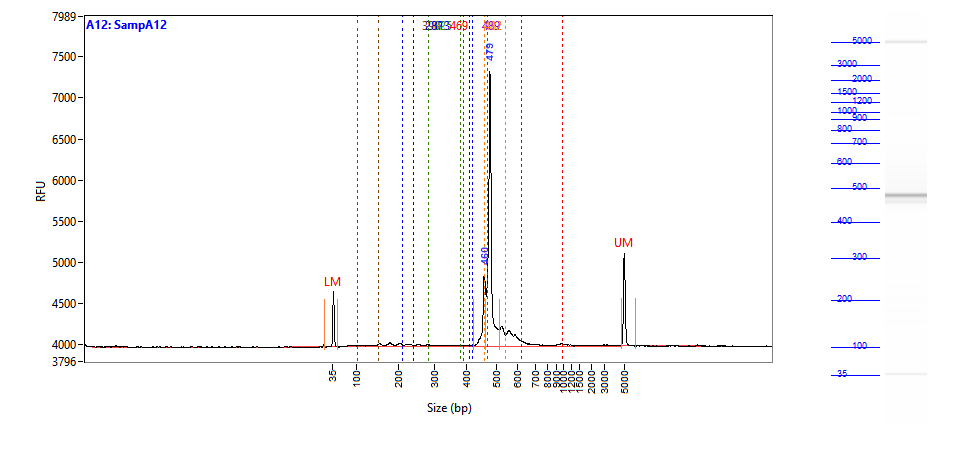

Supplement: Supplementary file 2 [file Data_Sheet_1.ZIP › Supplementary material/16s/16s-TB3.BMP]

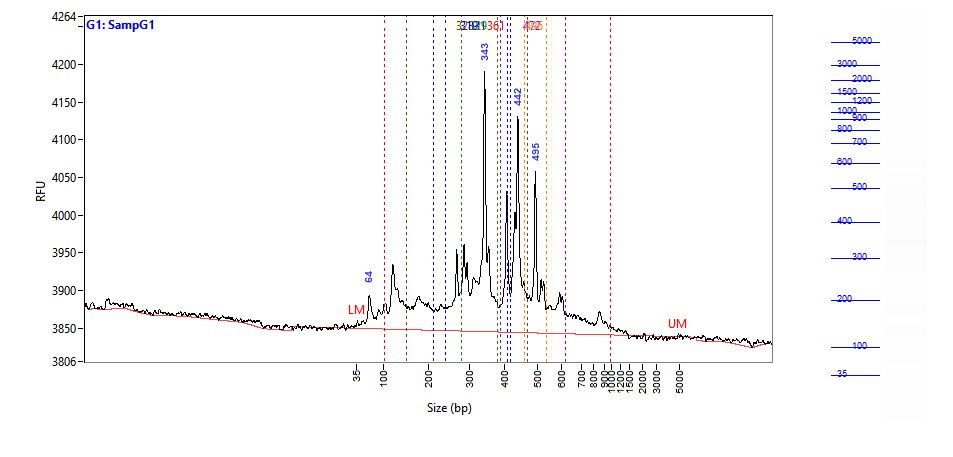

Supplement: Supplementary file 2 [file Data_Sheet_1.ZIP › Supplementary material/ITS/ITS-C1.JPEG]

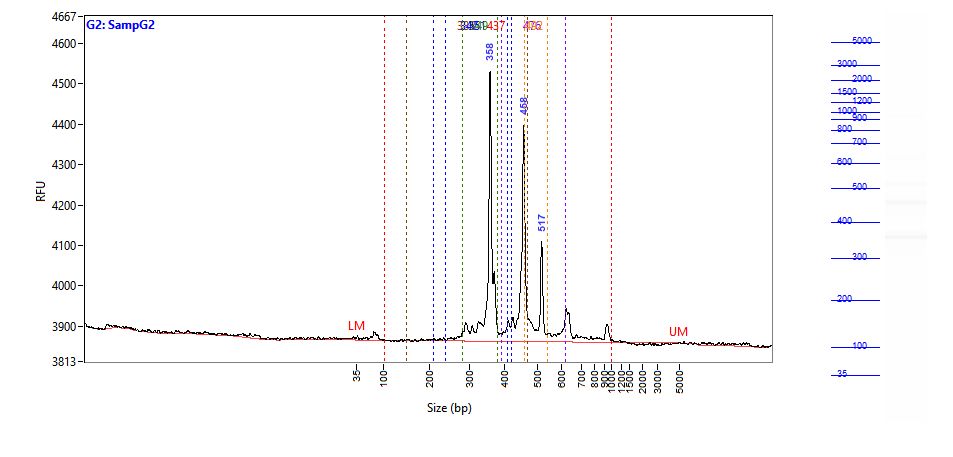

Supplement: Supplementary file 2 [file Data_Sheet_1.ZIP › Supplementary material/ITS/ITS-C2.JPEG]

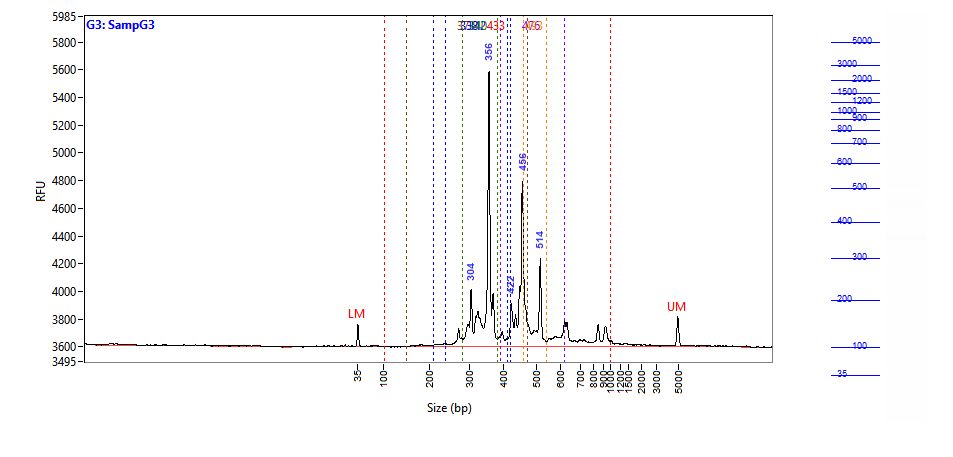

Supplement: Supplementary file 2 [file Data_Sheet_1.ZIP › Supplementary material/ITS/ITS-C3.JPEG]

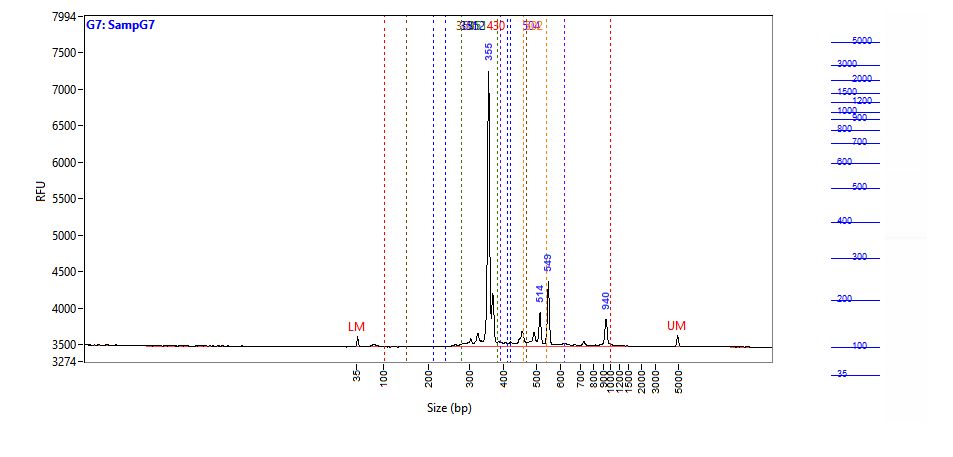

Supplement: Supplementary file 2 [file Data_Sheet_1.ZIP › Supplementary material/ITS/ITS-LB1.JPEG]

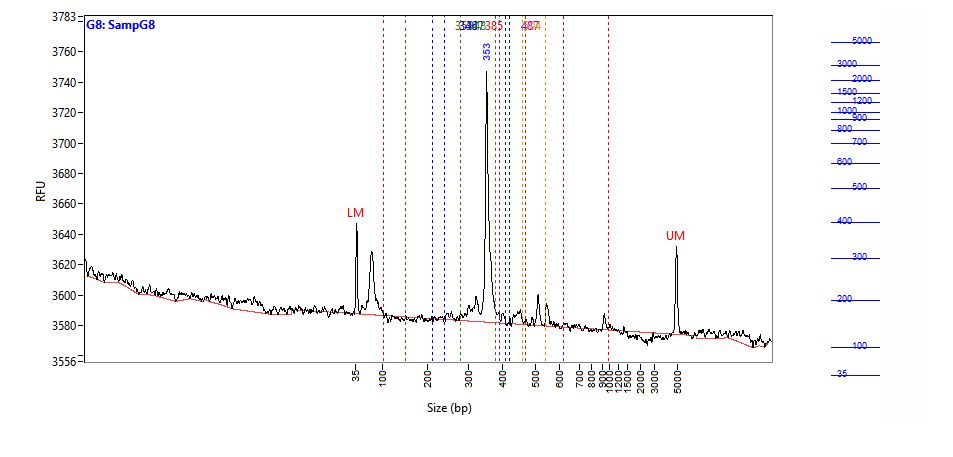

Supplement: Supplementary file 2 [file Data_Sheet_1.ZIP › Supplementary material/ITS/ITS-LB2.JPEG]

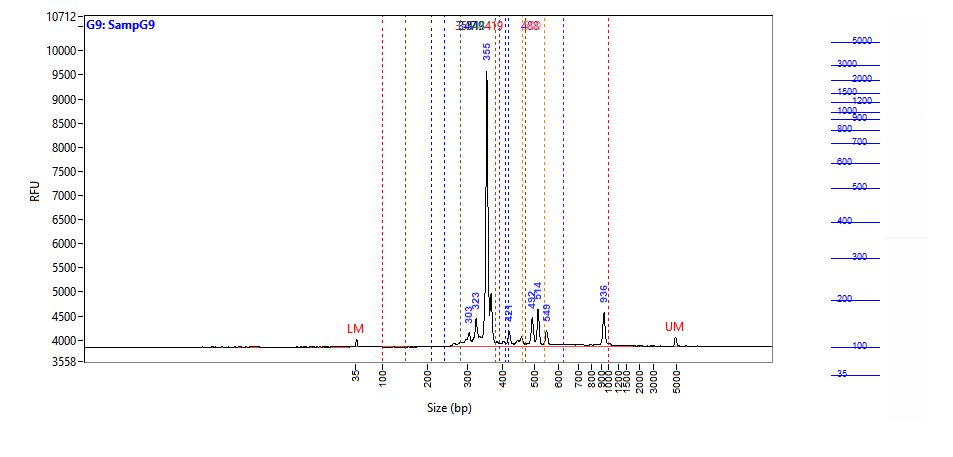

Supplement: Supplementary file 2 [file Data_Sheet_1.ZIP › Supplementary material/ITS/ITS-LB3.JPEG]

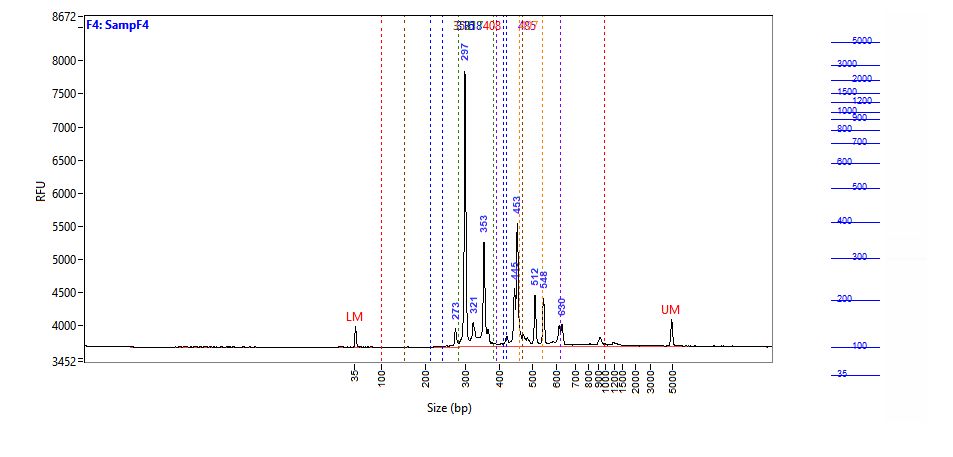

Supplement: Supplementary file 2 [file Data_Sheet_1.ZIP › Supplementary material/ITS/ITS-PB1.JPEG]

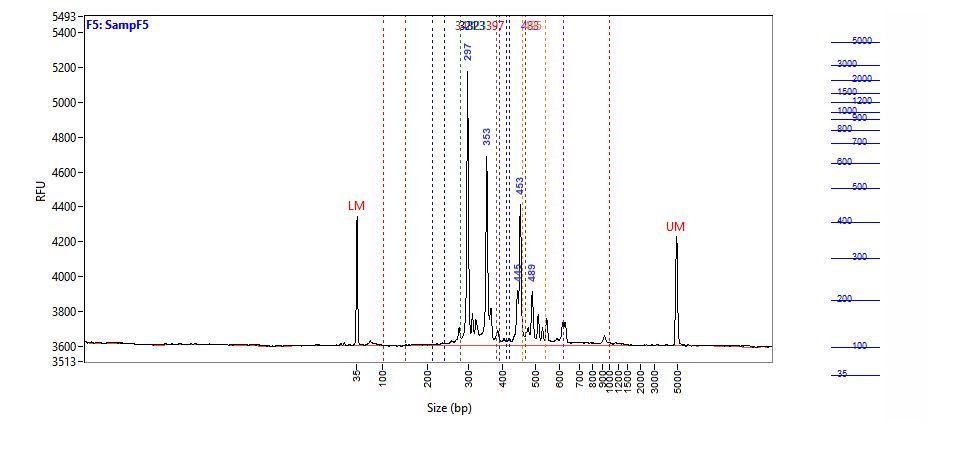

Supplement: Supplementary file 2 [file Data_Sheet_1.ZIP › Supplementary material/ITS/ITS-PB2.JPEG]

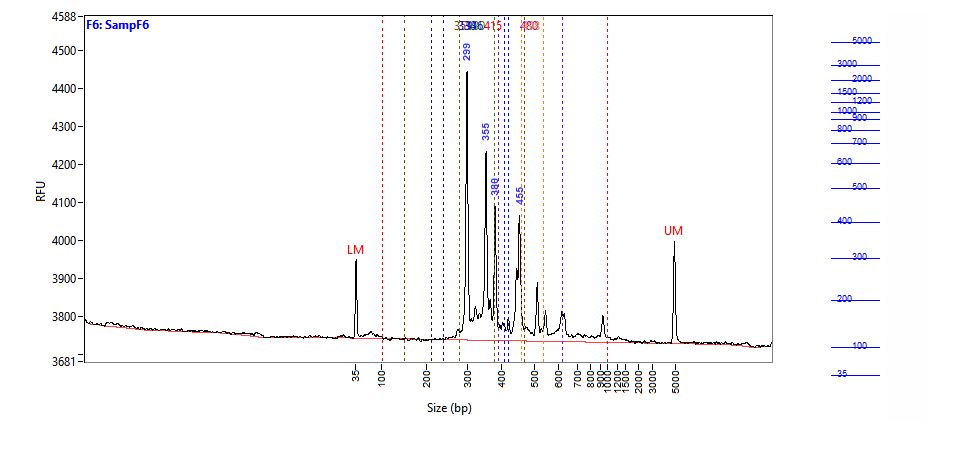

Supplement: Supplementary file 2 [file Data_Sheet_1.ZIP › Supplementary material/ITS/ITS-PB3.JPEG]

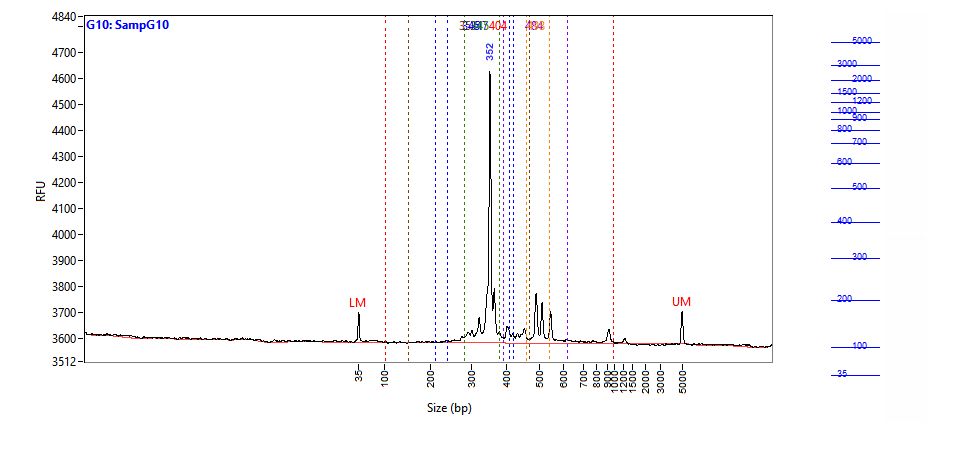

Supplement: Supplementary file 2 [file Data_Sheet_1.ZIP › Supplementary material/ITS/ITS-TB1.JPEG]

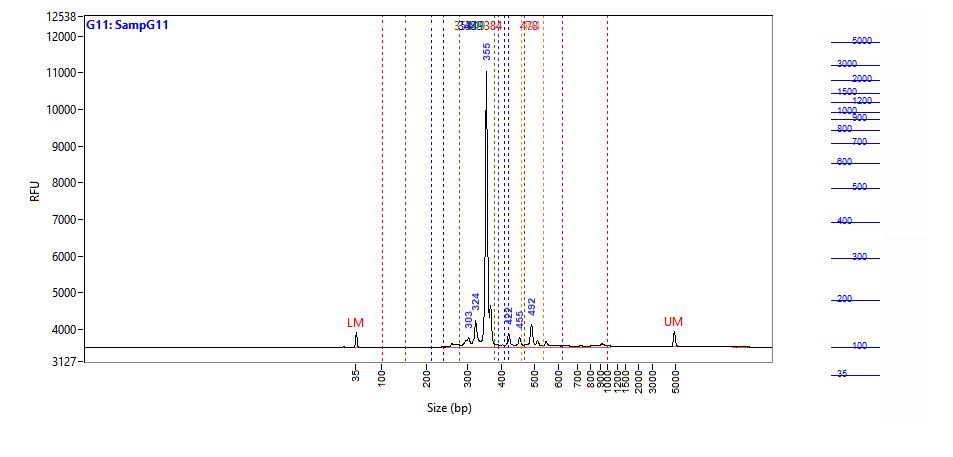

Supplement: Supplementary file 2 [file Data_Sheet_1.ZIP › Supplementary material/ITS/ITS-TB2.JPEG]

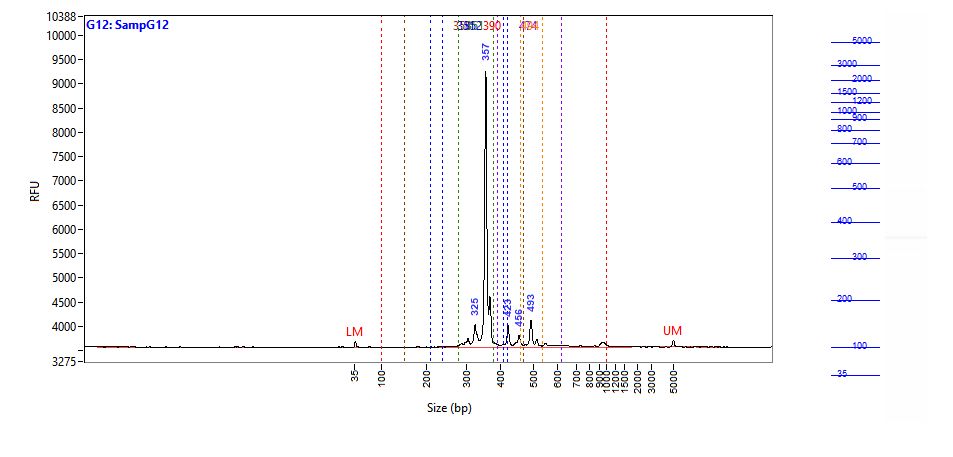

Supplement: Supplementary file 2 [file Data_Sheet_1.ZIP › Supplementary material/ITS/ITS-TB3.JPEG]
